# The high adaptive potential of *Abies alba* Mill. seedlings – biochemical and physiological studies of succession along the environmental gradient of a Cambrian quarry

**DOI:** 10.1186/s12870-025-06792-4

**Published:** 2025-07-02

**Authors:** Agnieszka Szuba, Ewelina Ratajczak, Tomasz Leski, Dominik Tomaszewski, Izabela Ratajczak, Gabriela Woźniak, Andrzej M. Jagodziński

**Affiliations:** 1 Institute of Dendrology, Polish Academy of Sciences, Parkowa 5, Kórnik, 62-035 Poland; 2 Department of Chemistry, Poznań University of Life Sciences, Wojska Polskiego 75, Poznań, 60-625 Poland; 3 Institute of Biology, Biotechnology and Environmental Protection, Faculty of Natural Sciences, University of Silesiain Katowice, Bankowa 12, Katowice, 40-032 Poland

**Keywords:** Succession, Quarry, In situ analyses, Acclimatization, Mycorrhiza, *Abies alb*a seedlings, Biochemical status, Stress symptoms, Antioxidant activity, Nonstructural carbohydrates

## Abstract

*Abies alba* Mill. (silver fir) needs specific soil and humidity conditions and seedlings are vulnerable to climatic extremes. Surprisingly, successful seedling establishment has been observed in disturbed habitats like active quarries. We compared 2-year old fir seedlings in three habitats—natural fir forest, disturbed forest, and exposed quarry—to explore the biochemical features that help them endure the first stages of succession in harsh environments. We assessed a range of parameters including reactive oxygen species (ROS) levels, antioxidant activity (2,2-diphenyl-1-picrylhydrazyl reduction), foliar pigments (chlorophylls and carotenoids), C and N contents, nonstructural carbohydrates, phenolics, and cell wall components analyzed via Fourier Transform Infrared (FTIR) spectroscopy, as well as ectomycorrhizal colonization and diversity. The analysis indicated that the fir seedlings were in unexpectedly good physiological condition despite the environmental constraints. Continuous exposure to harsh conditions (stony soils poor in C and N, extreme insolation, potential drought/flooding stress, etc.) was only slightly associated with ROS and antioxidant levels, roots of seedlings were fully mycorrhized, and their roots did not express signals of severe oxidative stress. Only a few seedling features clearly followed the environmental gradient; C (%), ectomycorrhizal fungal richness in roots, and total antioxidant content in stems decreased in harsh environments. Despite the lower chlorophyll levels, seedlings from the quarry had no decrease in C or N foliar levels. These firs did not have impaired N- or C-compound levels. Quarry seedlings had the highest nonstructural carbohydrates in needles and roots, protective foliar shifts (more carotenoids), and stronger stems (more structural carbohydrates, especially lignin). These findings demonstrate strong acclimatization capacity of *A. alba* seedlings and suggest the existence of stabilizing physiological mechanisms supporting survival in disturbed environments.

**Supplementary Information:**

The online version contains supplementary material available at 10.1186/s12870-025-06792-4.

## Introduction

*Abies alba* Mill., commonly known as silver fir, is a late-successional coniferous species widely distributed in the highlands and mountains of south-central Europe [1]. This species thrives in mesic conditions with deep soils, supported by a robust root system that allows it to tolerate episodic droughts [2]. However, silver fir is highly sensitive to specific environmental requirements, such as humus-rich topsoil and sufficient air humidity, making it vulnerable to climatic extremes during its early developmental stages, including drought, excessive frost, insolation, and late frosts [3]. Despite these limitations, silver fir demonstrates exceptional shade tolerance, enabling the formation of dense canopies under optimal growth conditions [4]. Furthermore, Walder et al. [5] show that *A. alba* thrives even under dry Mediterranean conditions in central Italy, suggesting that it may be more drought-resilient than traditionally understood.

Environmental changes and human activities significantly alter the habitats of silver fir, influencing its physiological responses and growth. Quarrying, for instance, drastically transforms landscapes by creating poor, oligotrophic mineral soils with extreme physical and chemical characteristics, such as high insolation, low organic content, and water stress risks [6, 7]. These conditions are typically unsuitable for climax species such as silver fir. However, spontaneous succession in abandoned or active quarries has shown remarkable regenerative potential, with quarries serving as biodiversity hotspots that transform over time into refuges for various plant and animal species [8]. Successional processes in such environments are often dominated by pioneer species like birch and pine, which are better adapted to the extreme conditions [9]. The presence of silver fir in such settings is particularly surprising, given its status as a climax species associated with stable, mature forest ecosystems [10].

Seedling growth and performance are influenced by environmental cues, initial seed resources, and genetic or epigenetic factors [11]. Parental environments also play a crucial role through maternal effects, which impact seed and seedling fitness, as well as epigenetic effects, where traits acquired by parent trees under stress can influence offspring responses [12]. Ectomycorrhizal fungi (ECM) are another critical factor of young seedling survival and functioning, enhancing nutrient uptake and improving stress resilience [13]. Silver fir, like most European temperate forest trees, relies on ECM associations for its survival and growth, particularly in disturbed environments with limited nutrient availability [14, 15]. High ECM fungal diversity has been reported in mature forests and naturally regenerated silver fir stands [16, 17], suggesting their essential role in colonizing degraded habitats.

Abiotic stress in plants, such as that experienced in quarry habitats, often results in the overproduction of reactive oxygen species (ROS), which can damage cellular structures [18]. To mitigate these effects, plants have evolved antioxidant defense mechanisms that maintain cellular homeostasis [19]. However, prolonged stress may lead to metabolic disruptions and reduced plant fitness [20, 21]. Studies have shown that plants exposed to harsh environments may develop stabilizing mechanisms over time, reducing the intensity of stress symptoms and enabling survival [22, 23]. The ability of silver fir seedlings to colonize quarries raises questions about their physiological and biochemical mechanisms for surviving and thriving in such extreme conditions.

To address these questions, we hypothesized that (i) silver fir seedlings growing in quarry habitats develop specific biochemical adaptations, such as enhanced antioxidant activity and shifts in photosynthetic pigments, to tolerate environmental stresses; (ii) ECM fungi play a critical role in facilitating nutrient acquisition and improving stress tolerance in these seedlings; and (iii) silver fir exhibits high metabolic plasticity, including changes in carbohydrate and nitrogen metabolism, supporting its acclimatization and survival along the environmental gradient from natural forests to quarries.

In our study site, these extreme quarry conditions are reflected in measurable abiotic indicators, including 3–5-fold higher insolation levels than in forested habitats, low organic matter and water-holding capacity, and shallow, rocky soils with minimal humus layer development.

This study investigated the physiological and biochemical responses of silver fir seedlings in three distinct habitats: natural forest, disturbed forest, and quarry. By analyzing stress and metabolic markers such as oxidative stress markers, antioxidant defences, carbohydrate and nitrogen metabolism, and ECM fungal colonization, we sought to understand the mechanisms underlying the survival of *A. alba* in suboptimal environments. To the best of our knowledge, this is the first comprehensive biochemical study, including analyses of needles, roots and frequently omitted stems of juvenile silver fir seedlings naturally growing in disturbed quarry habitats during early ecological succession, offering valuable insights into their acclimatization potential and adaptive strategies.

## Materials and methods

### Characteristics of the study area, experimental design, and plant material harvesting

**Fig. 1:**
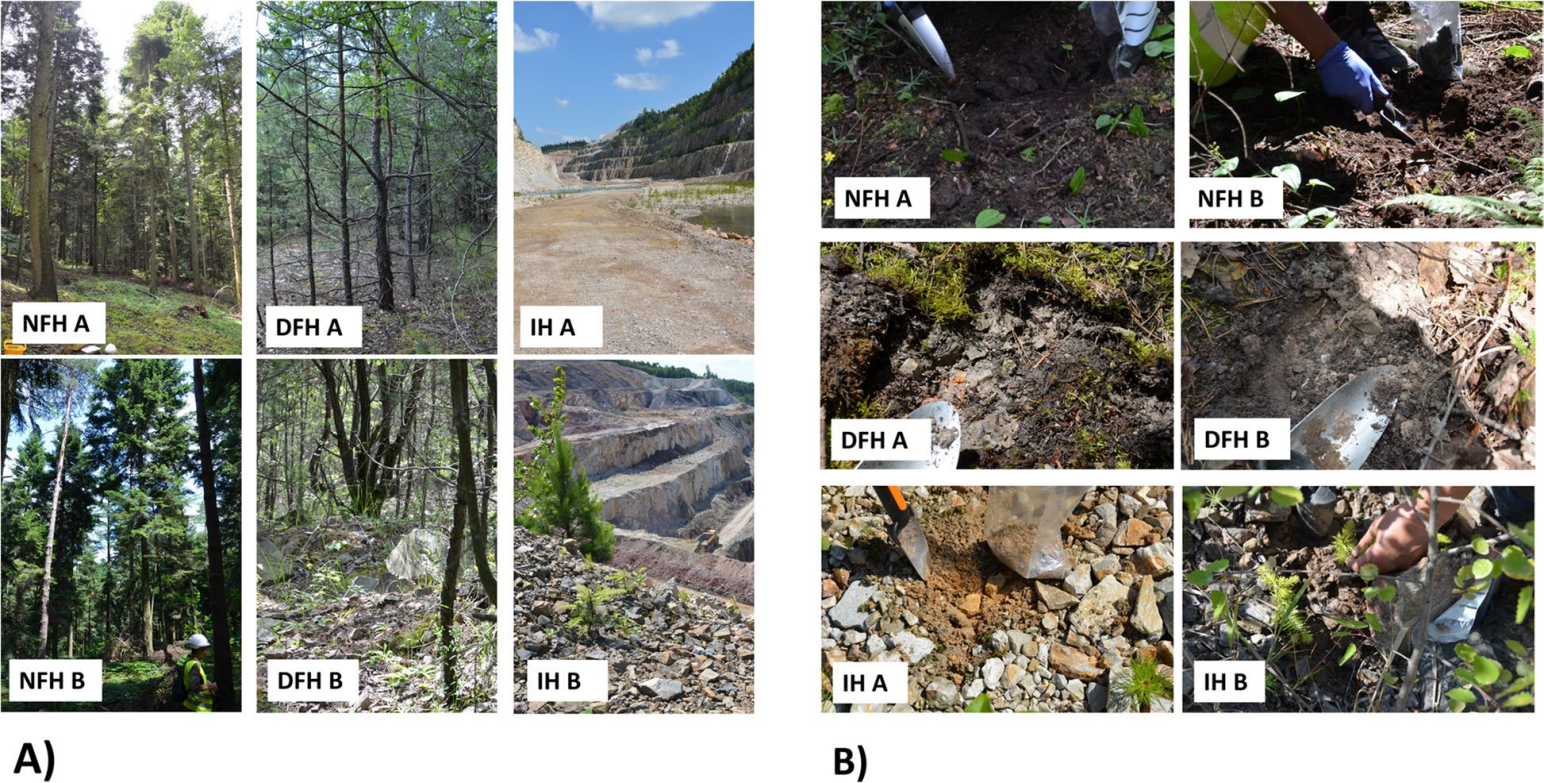
Representative images of all analyzed habitats (**A**). A representative close-up of the soil during sampling is shown, revealing the in situ soil structure (**B**). NFH – natural forest habitat; DFH – disturbed forest habitat; IH – initial habitat (for description, please see M&M section)

The present study area is located in central-eastern Poland in the Małopolska Upland (Kielce Upland microregion) within the Świętokrzyskie Mountains mesoregion. The study was performed in and surrounding the area of the aggregate quarry. Seedlings of A. alba from three distinct habitats were analyzed: (1) the Natural Forest Habitat (NFH), representing the natural environment of firs—an approximately 100-year-old fir forest with mature fir trees; (2) the Disturbed Forest Habitat (DFH), a dense young pine forest dominated by~15-year-old Scots pines growing on stony ground, considered an intermediate stage between the natural and initial habitats; and (3) the Initial Habitats (IHs), corresponding to the area of active excavation (Fig. 1A; for a detailed description of the habitats, please see Supplementary File 1). The abundance and density of fir seedlings were considerably greater in NFH habitats than in disturbed habitats (Supplementary File 1). The ground in the fir forest had humus-rich topsoil; in the pine forest, the topsoil was rocky with pockets of clay; and in the excavation area, there was bedrock with stones and only a few small patches of primary soil at the beginning of the soil formation process (Fig. 1B).

Due to the limited distribution of the *Abietetum polonicum* forest stands, the studied *A. alba* seedling individuals are the offspring of the local population from NFHs. The study was conducted in duplicate, referred to hereafter as A and B (Fig. 1), both of which included the three seedling habitats (NFH, DFH and IH). Each duplicate (A and B) refers to an autonomous study of analysed habitat. Plots A and B were a minimum of 100 m apart. Independent, duplicated variants were analyzed (statistically) separately. Therefore, six plots were studied and grouped into three disturbance-level habitats: NFH, DFH and IH. From all 6 analyzed sites, the soil samples and fir seedlings were collected at the same time, during the middle of the growing season, in early July 2020.

Two-year-old seedlings were collected for biochemical analysis (minimum of 10 seedlings per plot), immediately frozen in liquid nitrogen, and stored at −80 °C until analysis. In the stony DFH and IF soil, a complete harvesting of the entire root system was impossible, making typical biometric analyses such as root: shoot ratio unachievable. For this reason biometric parameters were limited to stem length analysis (made using electronic calipers ± 1 mm).

The formal identification of the samples used in this study was performed by prof. Andrzej M. Jagodzinski from the Institute of Dendrology Polish Academy of Sciences. No herbarium specimens were collected. For each pooled sample, fragments of a minimum of 20 randomly collected needles or fragments of stems and roots were collected from at least 5 randomly selected seedlings. The sampling procedure was optimized during a previous field biochemical study [24].

The study area is located at an elevation of approximately 310–350 m above sea level. According to regional climate data for the years 2001–2020, the mean annual temperature at the site was 8.7 °C, and the average annual precipitation was approximately 702 mm. The region has a temperate climate with moderately cold winters and warm summers.

The studied quarry is located on rare Cambrian sandstone, forming a gradient of habitat disturbance. This substrate creates harsh conditions due to low pH, poor water retention, and minimal organic matter. In freshly excavated areas (IHs), primary succession begins with scattered pioneer species and frequent *A. alba* seedlings. Older sites (ca. 60 years post-mining) show secondary succession (DFHs), with conifer patches and spontaneous *A. alba* regeneration. Due to the rarity of such substrates in temperate Europe, comparable ecological data remain limited (Supplementary File S1).

### Soil analysis

Soil samples (vol 1 dm3) were collected at depths of 0–20 cm after removal of the sediment and surface layer (with dead plant remnants, etc.) from at least 10 locations in the vicinity of *A. alba* seedlings grown on a particular plot and immediately secured at 4 °C until analysis. To evaluate the parameters of the rhizosphere conditions, several biochemical soil features were evaluated. The soil dry matter percentage was analyzed after drying the soil samples at 60 °C for 48 h according to the formula [DM (%) = (fresh soil weight/dry soil weight) × 100%] (Supplementary File S1). The soil pH of the fir rhizosphere soil samples was analyzed in dry, sifted soil samples (50 mg). The soil was resuspended in Milli-Q water, agitated for 10 min at 4 °C, and filtered through a Miracloth (Millipore), after which the soil pH_H2O_ was analyzed in the obtained supernatants (*n* = 6) using an Elmetron CX-551 pH meter. The electrical conductivity (EC) (substrate-to-solution ratio 1:2.5) was assessed according to Bednarek et al. [25]. The measurements were performed by the potentiometric method using an SEN 81 st TI X electrode. The substrate organic carbon content was estimated in the collected samples by loss on ignition (LOI) analysis. LOI was measured by burning 5–10 g of soil (dried at 105 °C to constant weight) in a muffle furnace at 550 °C for 5 h [26, 27]. Total N was estimated using the Kjeldahl method [25]. The water holding capacity (WHC) of the substrate was measured by the gravimetric method [28].

The nonspecific dehydrogenase activity (DHA) was evaluated using a method based on the reduction of 2,3,5-triphenyltetrazolium chloride (TTC) to triphenyl formazan (TPF) [23]. The fresh soil samples (5 g, *n*= 8) were incubated at 30 °C for 24 h in 1% TTC solution. After incubation, the TPF form was extracted with 25 ml of 96% ethanol and measured spectrophotometrically at 480 nm with a Beckman Coulter DU-640 UVVIS (Beckman Instruments, Inc., Fullerton, CA, USA). The results are expressed as nmol TPF g^−1^ dry soil h^−1^.

### Measurement of light conditions

Within each plot, we measured diffuse light availability using an LAI-2070 device (LI-COR, Inc., Lincoln, NE; http://www.licor.com). As a measure of light availability, we used diffuse noninterceptance (DIFN, dimensionless), the ratio of photon flux density in the study plot, and a reference plot (open-sky area located near the study plots), as well as the leaf area index (LAI). Within each plot, we sampled 6 series of 5 measurements at randomly selected points at a height of 0.5 m. Light condition measurements were taken during the time of maximum canopy development to account for the maximum light availability limitation (cf. [29–31]).

**Fig. 2:**
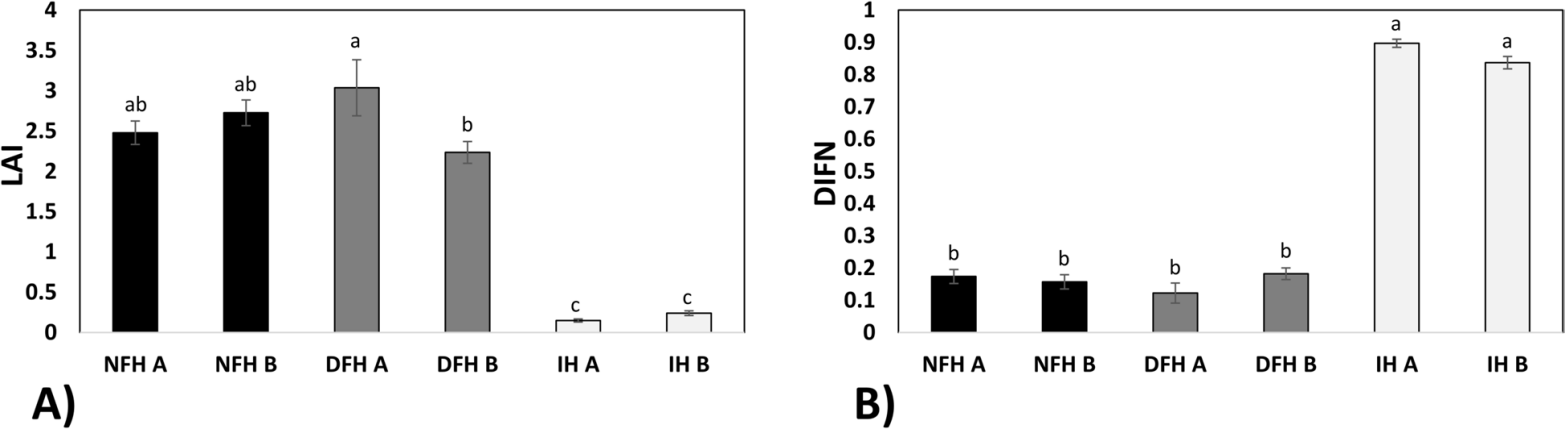
Light conditions. Intensity of insolation for affected fir seedlings. LAI (**A**) and diffuse noninterceptance (DIFN) (**B**) were calculated for particular plots. NFH – natural forest habitat; DFH – disturbed forest habitat; IH – initial habitat (for description, please see M&M section). Different letters indicate significant differences according to the HSD post hoc test (*n* = 6)

The analyzed fir seedlings grew naturally on the forest floor, and the level of available light was limited by the surrounding vegetation. Similarly, the foliage barrier created by plants growing above the fir seedlings was similar for NFH and DFH and much lower for IH (Fig. 2A). The detailed analysis of DIFN values defining parts of the ground surface not covered by plants revealed that the excavation area was (as expected) indeed characterized by significantly greater insolation (3 to 5 times greater) than that in the fir forest, whereas the light available for seedlings from natural regeneration did not differ among the four NFH and DFH plots (Fig. 2B).

### Root colonization analysis

For the ectomycorrhizal (ECM) evaluation, 10 seedlings were taken from each study plot. The roots of the seedlings were extracted from the soil, washed with tap water, and cut into 4–5 cm fragments. All the fine roots from each seedling were counted and examined for ECM colonization using a dissecting microscope at a magnification of 10–60 ×. Ectomycorrhizas were recognized based on specific characteristics: the presence of a fungal mantle, external hyphae, or rhizomorphs, as well as the absence of root hairs. The level of ECM colonization was expressed as a percentage of the total number of fine roots that exhibited ECM associations out of the overall number of fine roots examined. Ectomycorrhizas were further categorized into individual morphotypes based on their morphological characteristics using Agerer’s Colour atlas of ectomycorrhizae (1987–2008) as the reference [32].

### Needle surface analysis by scanning electron microscopy

Photographs taken with a scanning electron microscope (SEM) were used to analyze the needle surfaces. Two needles from three individuals were selected for each of the six plots. The needles were obtained from naturally collected material that was immediately frozen and stored at a temperature of −20 °C. Before observation under the microscope, the material (needle fragments) was dried at room temperature and coated with gold and palladium. The samples were observed using a secondary electron (SE) detector at a voltage of 20 kV. A series of photographs at various magnifications were obtained from each needle.

### Needle pigments

Needle pigments, including chlorophyll (a), chlorophyll (b), and the fractions of carotenoids (chlorophyll (c + x)), were analyzed in pooled needle samples. A total of 20 mg (FW) of the needle samples (*n* = 6) was used for the analysis. The pigments were extracted using 80% (v/v) acetone as described by Lichtenthaler and Wellburn [33]. To measure the absorbance of the extracted pigments, a Beckman Coulter DU-640 UV‒Vis spectrophotometer from Beckman Instruments, Inc., Fullerton, CA, USA, was used. The absorbance was measured at wavelengths of 470 nm, 646 nm, and 663 nm. The needle pigment concentrations were expressed in fresh weight.

### Elemental analysis of C and N

Total nitrogen and carbon were determined in dried and ground pooled samples of needles, stems, and roots of seedlings (*n* = 3). Twenty mg (per sample) of dry biological material was analyzed with a CHNS analyzer (2400 CHNS/O Series II System, PerkinElmer, Waltham, MA, USA), after which the C/N ratio was calculated.

### Determination of soluble total nonstructural carbohydrates and starch

Soluble nonstructural carbohydrates (SCs) and starch were extracted from needle, stem, and root samples of fir seedlings using a previously described standard colorimetric assay [24]. The leaves were dried at 60 °C for 72 h to remove moisture. SCs were extracted at room temperature using a mixture of methanol, chloroform, and water (12:5:3, v/v/v). The polysaccharides in the extracted SCs were hydrolyzed into simple sugars in the presence of sulfuric acid. The resulting monosaccharides were measured by reacting them with anthrone and measuring the absorbance at 625 nm, with glucose serving as a standard. To determine the residual starch content, the starch was first hydrolyzed into glucose using α-amylase-glucosidase. The concentration of glucose was then measured by reacting it with glucose oxidase–peroxidase o-dianisidine dihydrochloride reagent and measuring the absorbance at 450 nm, with glucose again used as a standard. The concentrations of SC and starch were normalized to those of DW.

### Estimation of the structural carbohydrate level

Fourier transform infrared spectroscopy (FTIR) was used to estimate the composition of the cell wall (CW) components, including the major carbohydrates cellulose, hemicellulose and lignin, in the roots, stems, and needles of the fir seedlings. FTIR analysis was performed following the methods of Pandey and Nagveni [34].

For the analysis, dry samples of roots, stems and needles were pooled and ground into a powder. The pooled sample powder (*n* = 3) was then mixed with KBr at a ratio of 1/200 mg. The spectra were registered using an FTIR Nicolet iS5 Thermo instrument with Fourier transformation in the range from 600 to 4000 cm^−1^ at a resolution of 2 cm^−1^, registering 16 scans. Basic spectra were used to conduct FTIR analyses of the samples. The spectral bands characteristic of lignin (namely, at 1640 cm^−1^ (bending the H–O–H orbital of absorbed water and skeletal aromatic rings), at 1515 cm^−1^ (skeletal aromatic rings) and 1320 cm^−1^ (stretching C–O and guaiacol rings)) as well as those characteristic of celluloses and hemicelluloses (namely, at 1735 (associated with C-O stretching vibrations in unconjugated ketones, carboxyl groups, and ester groups, predominantly found in hemicelluloses), at 1375 cm^−1^ (deformation of C–H in cellulose and hemicellulose) and at 1160 cm^−1^ (vibration of C–O–C in cellulose and hemicelluloses)) were analyzed [35, 36]. Wavenumbers at 1375 cm^−1^ and at 1515 cm^−1^ were selected for cellulose and hemicellulose: lignin ratio calculations.

### Determination of total phenolic compounds

To analyze the total soluble phenols in the fir needles, a standard colorimetric assay was employed following a previously described method [37]. Phenolic compounds were extracted from the dried and ground needles, stems, and roots of seedlings through a two-step process. First, the leaves were boiled in 96% ethanol and then in 80% ethanol for 15 min each. The combined extracts, in a Na_2_CO_3_ environment, were utilized to determine the total phenol content. The reactions of the extracts with the Folin-Ciocalteu phenol reagent were carried out, and the absorbance was measured at 660 nm. The results of the analysis are expressed as micromoles of chlorogenic acid per gram of dry weight. Chlorogenic acid is used as a standard to quantify the phenol content in leaves.

### Oxidative stress and tolerance indicators

#### Release of Hydrogen Peroxide (H_2_O_2_)

Samples of needles, stems and roots (1 g) were homogenized in 5% TCA. The homogenate was then centrifuged for 20 min at 4 °C. The ferrithiocyanate method was used to determine the H_2_O_2_ level [38]. The reaction mixture contained 0.5 ml of extract, 1.5 ml of 50% TCA, 0.4 ml of 10 mM ferric ammonium sulfate, and 0.2 ml of 2.5 M potassium thiocyanate. The control contained 0.5 ml of 5% TCA instead of the extract. The absorbance was measured spectrophotometrically (Shimadzu UV-2501PC) at 480 nm. The amount of H_2_O_2_ was determined from the previously prepared standard curve and normalized to the fresh weight.

#### Total antioxidant capacity (TAC)

The TAC was determined using the reduction of 2,2-diphenyl-1-picrylhydrazyl (DPPH) following the method described by Molyneux [39]. Tissues were homogenized in 0.5 ml of 100% (v/v) methanol. The homogenates were centrifuged at 7 000 × g for 10 min at 4 °C, after which 20 μl of the extract was added to 180 μl of 120 μM DPPH dissolved in methanol on 96-well plates. The reaction mixture was incubated for 15 min in the dark at room temperature. The concentration of reduced DPPH was measured at 517 nm using a microplate reader. The antioxidant capacity was expressed as the reduction of DPPH and calculated using the formula (A_0_ − A_s_)/A_0_ × 100%, where A_0_ represents the absorbance of the blank and A_s_ represents the absorbance of the sample. This calculation allowed us to quantify the percentage reduction in DPPH activity caused by the antioxidants in the sample, reflecting their antioxidant capacity.

#### Determination of malondialdehyde (MDA)

The MDA content, a reliable marker commonly used to assess the extent of damage caused by peroxidation of polyunsaturated fatty acids in stressed plants [40], was determined in needle, stem and root samples by reaction with thiobarbituric acid (TBA) as described by [41]. The samples were suspended in 5% TBA in 20% trichloroacetic acid (TCA) and incubated in a boiling water bath for 20 min. Thereafter, the samples were immediately cooled on ice to stop the reaction. The absorbances at 532 and 600 nm were determined. The MDA concentration was estimated by subtracting the nonspecific absorption at 600 nm from the absorption at 532 nm. The amount of MDA was calculated by taking into account the millimolar extinction coefficient of the MDA-TBA complex, which is 1.55 mM^−1^ cm^−1^ and was adjusted according to the nonspecific absorption at 600 nm. The concentration of MDA is expressed in nmol/mg fresh weight.

### Statistical analyses

The biometric and biochemical results were analyzed using JMP Pro 13.0.0 software (SAS Institute, Inc.). After checking the normality and homogeneity of the data using the Shapiro–Wilk test and Levene’s test, respectively, analysis of variance (one-way ANOVA, α = 0.05) was used to compare all six plots, that is IH, DFH and NFH with their duplicates (the particular six plots were compared between each other). Means were compared and considered significant at *p* < 0.05 using Tukey’s honest significance post hoc test, if possible.

## Results

### Soil characteristics

**Fig. 3:**
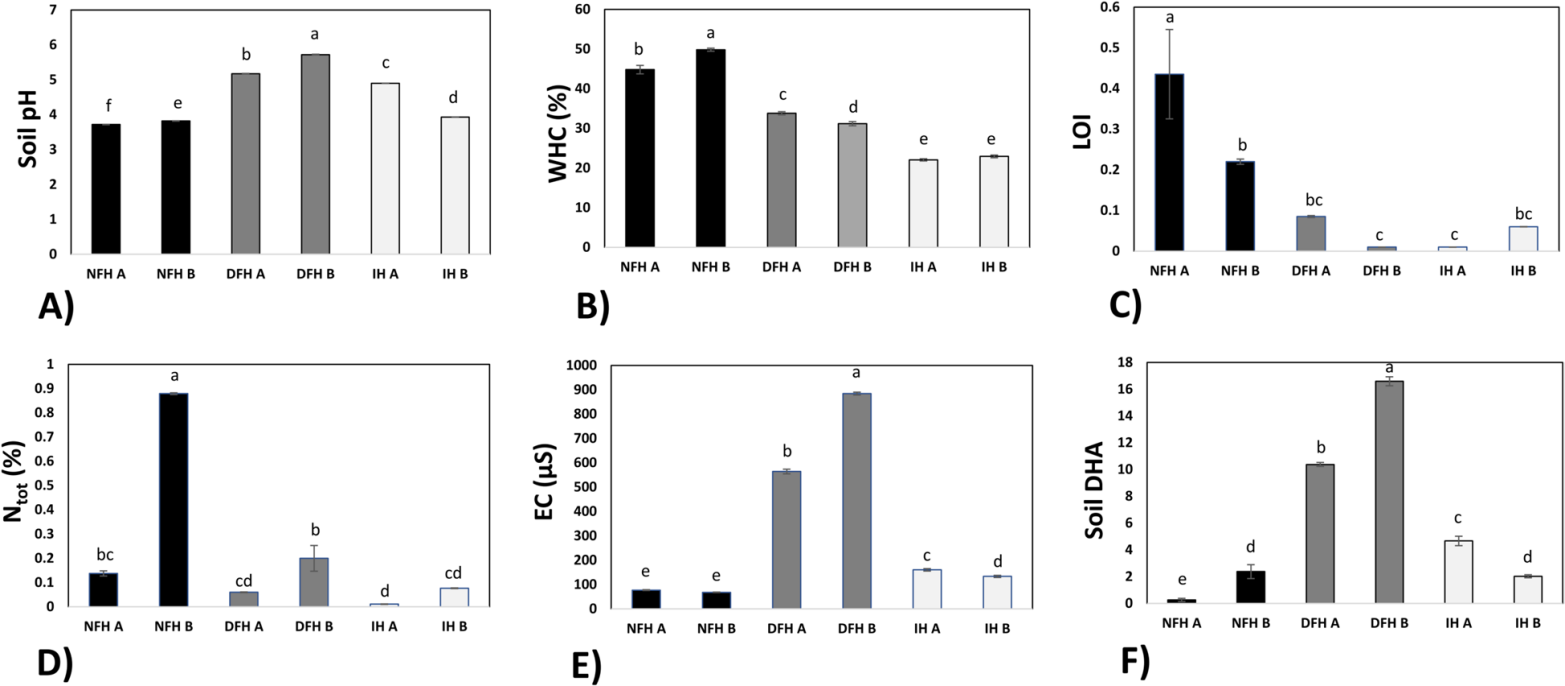
Soil biochemistry. Soil pH (**A**), soil water holding capacity (WHC) (%) (**B**), loss on ignition (LOI) (C), Ntot (%) (**D**), soil conductivity (EC) (**E**), and soil and nonspecific dehydrogenase activity (DHA) (**F**) were all measured in the soil collected from the rhizosphere of the analyzed fir seedlings. Values are expressed as the means ± SEs. Different letters indicate significant differences (*p* ≤ 0.05) according to an HSD test (for A, *n* = 6; for E, *n* = 8; for B-D, *n* = 12). NFH – natural forest habitat; DFH – disturbed forest habitat; IH – initial habitat (for description, please see M&M section)

One of the most significant differences between the analyzed habitats was related to soil characteristics. Soil parameters showed clear variation across habitats, with noticeable similarities between the two independent plots representing the same habitat (A vs. B) (Fig. 3). Dry matter (%) was consistent across most plots (81–92%), except for NFH A, where it was significantly lower (59%) (Supplementary File S1).

The soil in the mature fir forest (NFH) was more acidic compared to other habitats, while the DFH had less acidic soils, although still below neutral pH (Fig. 3A). Soil water holding capacity (WHC) decreased along the disturbance gradient: NFH → DFH → IH (Fig. 3B). Similarly, soil organic matter (LOI) content was significantly higher in NFH, with DFH and IH showing fluctuating but generally lower values (Fig. 3C). In contrast, nitrogen content [Ntot (%)] did not follow a clear gradient, although the highest levels were detected in NFH plots and the lowest in the IH at the bottom of the quarry (Fig. 3D).

Electrical conductivity (EC), a measure of dissolved elements in the soil solution, showed interesting patterns, being significantly greater in DFH than in IH, with the lowest values in NFH. Although statistically significant, the differences between NFH and IH were relatively small (Fig. 3E). Soil dehydrogenase activity (DHA), an indirect measure of microbial activity, revealed a different trend, closely following soil acidity. DHA was over five times higher in DFH than in NFH, while IH displayed intermediate values (Fig. 3F).

### Plant characteristics

**Fig. 4:**
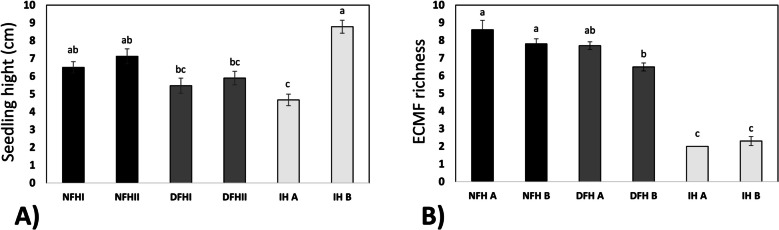
Seedling characteristics. Seedling height (**A**) and seedling root colonization (**B**). The average species richness of ectomycorrhizal fungi colonizing fir seedlings growing in three types of habitats. NFH – natural forest habitat, DFH – disturbed forest habitat, IH – initial habitat (for description, please see M&M section). The values are expressed as the means (*n* = 6) ± SEs. Different letters indicate significant differences (*p* ≤ 0.05) according to an HSD test

The analyzed fir seedlings did not exhibit a clear growth response to environmental pressure. The tallest seedlings were observed on the edge of the quarry (IH B), whereas the shortest seedlings were on its bottom. The seedlings growing in the optimal environmental condition of the fir forest (NFH) as well as young firs growing in the spots with intermediate environmental pressure were characterized by intermediate heights (Fig. 4A).

### Root ectomycorrhizal colonization

In all of the investigated habitats, the fine-root ECM colonization rate of the naturally regenerated *A. alba* seedlings was 100%. In total, 23 ECM fungal taxa were identified, 17 of which were in the NFH, 16 in the DFH, and 3 in the IH habitats. In contrast to the root colonization rate, the structure of the root colonization zone differed significantly between the analyzed habitats, clearly reflecting the increased initial character of the roots. The average ECM fungal taxa richness ranged between 2.3 in the IH B plot and 8.6 in the NFH A plot. Overall, the IH type was characterized by significantly lower species richness than the other two habitat types (Fig. 4B).

### Needle surface structure

**Fig. 5:**
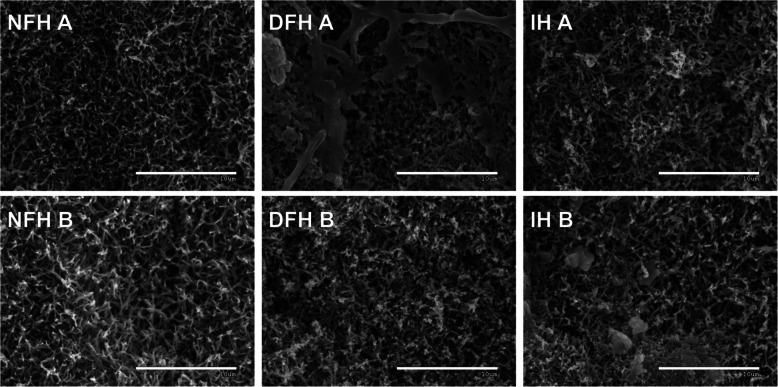
Needle waxes. Representative SEM images of wax structures on the abaxial surface of needles of fir seedlings from the analyzed plots. NFH – natural forest habitat; DFH – disturbed forest habitat; IH – initial habitat (for description, please see M&M section); scale bars = 10 μm

Stomata in *A. alba* are located on the abaxial side of the needles in two longitudinally arranged bands. Within these bands, the stomata are arranged in rows. The epidermis in which the stomata occur produces epicuticular wax in the form of tubes (tubules), which is typical for most gymnosperm species. The tubules are absent outside the stomatal zone, where the epidermis is covered with a smooth wax layer. The SEM analysis focused on analyzing the wax structures and the level of surface contamination of the needles. The samples were classified as weakly or moderately contaminated with mineral material (with the highest abundance of mineral contamination observed in IH B; Fig. 5). The presence of biological materials on the needles was limited by the occurrence of fungal hyphae from unidentified species and their spores. Their presence was sporadic, except for the needles from plot DFH A, where their mass was observed, along with strong local degradation of the wax structures associated with it (Fig. 5). Except for the abovementioned biotic and abiotic contamination, the microstructure of the epicuticular waxes (length, width and arrangement of tubes) did not vary significantly between the analyzed treatments, i.e., NFH, DFH and IH (Fig. 5).

### Needle pigments

**Fig. 6:**
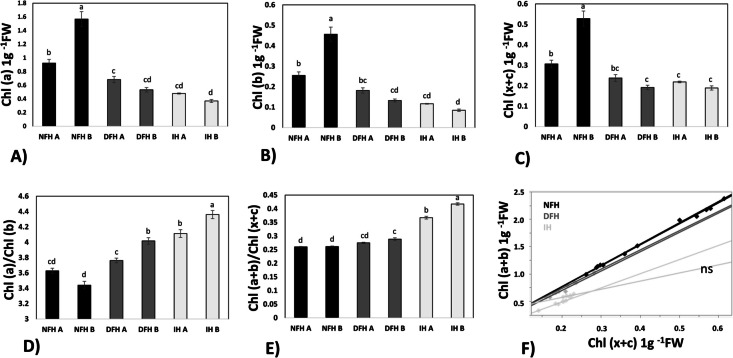
Chlorophyll (a) concentrations (**A**), chlorophyll (b) concentrations (**B**), xanthophyll and carotenoid (chlorophyll (x + c)) concentrations (**C**), chlorophyll (a): chlorophyll (b) ratios (**D**), chlorophyll (a + b): chlorophyll (x + c) ratios (**E**) and correlations (**F**) of the needle pigments. The values are expressed as the means (*n* = 6) ± SEs. Different letters indicate significant differences (*p* ≤ 0.05) according to an HSD test. FW – fresh weight; ns – not significant. NFH – natural forest habitat; DFH – disturbed forest habitat; IH – initial habitat

The highest concentrations of all foliar pigments, including chlorophylls (Fig. 6A and B) and carotenoids (Fig. 6C), were observed in the needles of seedlings grown in the natural fir forest, and the concentrations decreased in the order NFH → DFH → IH, with the lowest concentration occurring at the edge of the quarry (IH B). The extreme environment of the quarry (and, less significantly, the rocky young pine forest (DFH)) was reflected in the relationships between particular pigments: the more disturbed the fir seedling habitat was, the greater the chl (a): chl (b) ratio (Fig. 6D) and the greater ratios of carotenoids were detected than those of chlorophylls (Fig. 6E). These changes were reflected in the weakening of the relationship between chlorophylls (a + b) and carotenoids; these parameters were strongly positively correlated in the needles of seedlings grown in natural habitats but uncoupled in IH B (Fig. 6F).

### C- and N-compounds in seedlings

Total C (%) was highest in the needles of seedlings in natural habitats, with no significant differences between those in DFH and IH (Table 1). In stems, C (%) decreased progressively from NFH to DFH and IH, while roots showed significant differences with the lowest values in IH (Table 1). Nitrogen [Ntot (%)] was highest in DFH needles but greater in the needles and roots of IH seedlings compared to DFH (Table 1). Consequently, the C:N ratios were highest in DFH and lowest in NFH across whole seedlings (Table 1).

**Table 1:** Biochemical parameters of *Abies alba* seedlings analyzed

Feature	Seedling organ	NFH A	NFH B	DFH A	DFH B	IH A	IH B
**C (%)**	**Needle**	48.2 ± 0.05 ab	48.6 ± 0.13 a	47.5 ± 0.03 c	47.8 ± 0.06 bc	47.5 ± 0.17 c	47.5 ± 0.07 c
	**Stem**	47.9 ± 0.06 a	48.4 ± 0.21 a	46.4 ± 0.05 b	47.7 ± 0.33 a	45.9 ± 0.14 bc	45.3 ± 0.27 c
	**Root**	48.6 ± 0.18 a	47.0 ± 0.23 b	45.7 ± 0.05 c	46.2 ± 0.06 c	44.7 ± 0.15 d	43.4 ± 0.02 e
***N* (%)**	**Needle**	1.02 ± 0.003 ab	1.23 ± 0.010 a	0.62 ± 0.015 c	0.59 ± 0.012 c	0.96 ± 0.151 ab	0.75 ± 0.021 bc
	**Stem**	0.79 ± 0.041 a	0.56 ± 0.009 b	0.42 ± 0.020 b	0.49 ± 0.077 b	0.49 ± 0.037 b	0.49 ± 0.026 b
	**Root**	0.66 ± 0.006 bc	0.78 ± 0.046 ab	0.36 ± 0.023 d	0.37 ± 0.020 d	0.97 ± 0.091 a	0.53 ± 0.041 cd
**C:N ratio**	**Needle**	55.0 ± 0.32 d	46.0 ± 0.41 d	89.6 ± 1.89 ab	94.9 ± 1.65 a	60.2 ± 8.53 cd	74.0 ± 1.87 bc
	**Stem**	70.8 ± 3.44 b	100 ± 2.22 ab	129 ± 6.23 a	119 ± 16.58 a	110 ± 9.22 ab	109 ± 6.70 ab
	**Root**	85.9 ± 0.75 bc	70.5 ± 4.05 bc	149 ± 10.2 a	145 ± 8.5 a	54.6 ± 4.70 c	95.9 ± 6.96 b
**SC (mg g**^**−1**^**DW)**	**Needle**	103 ± 1.7 a	100 ± 1.7 ab	88.9 ± 1.56 c	94.0 ± 1.19 bc	107 ± 1.9 a	108 ± 2.1 a
	**Stem**	42.0 ± 1.60 a	20.7 ± 0.26 c	29.3 ± 0.82 b	26.6 ± 0.49 b	39.9 ± 1.15 a	39.1 ± 1.30 a
	**Root**	43.50 ± 0.93 b	35.10 ± 0.87 c	29.70 ± 0.81 cd	28.50 ± 1.70 d	62.80 ± 1.91 a	41.50 ± 0.93 b
**Starch (mg g**^**−1**^**DW)**	**Needle**	35.2 ± 0.47 e	20.1 ± 0.43 f	68.4 ± 0.94 d	79.4 ± 0.86 c	142 ± 1.58 a	115 ± 2.65 b
	**Stem**	28.1 ± 1.33 d	11.8 ± 0.26 f	76.3 ± 0.83 a	18.5 ± 0.34 e	51.4 ± 1.82 b	42.2 ± 1.07 c
	**Root**	44.7 ± 0.51 d	56.7 ± 0.88 c	127 ± 2.9 a	69.1 ± 1.29 b	41.4 ± 1.66 d	63.7 ± 1.58 bc
**Structural carbohydrates (A1375/1515 FTIR ratio)**^**a**^	**Needle (ns)**^**b**^	1.56 ± 0.121	1.63 ± 0.309	1.25 ± 0.091	1.26 ± 0.036	1.35 ± 0.184	1.55 ± 0.261
	**Stem**	0.483 ± 0.071 d	0.630 0.003 cd	0.743 ± 0.056 bc	0.600 ± 0.026 cd	1.071 ± 0.009 a	0.882 ± 0.050 ab
	**Root (ns)**^**b**^	0.685 ± 0.014	1.069 ± 0.144	0.825 ± 0.282	0.929 ± 0.006	0.908 ± 0.054	1.019 ± 0.006
**Phenolics (µmol CGA g**^**−1**^** DW)**	**Needle**	373 ± 9.5 d	378 ± 5.4 d	561 ± 28.5 c	847 ± 14.1 a	323 ± 8.7 d	647 ± 13.2 b
	**Stem**	244 ± 5.2 abc	199 ± 10.0 cd	290 ± 18.0 a	265 ± 16.4 ab	176 ± 11.5 d	212 ± 13.3 bcd
	**Root**	121 ± 13.3 c	148 ± 15.9 bc	244 ± 4.1 a	237 ± 6.3 a	170 ± 4.6 b	147 ± 5.8 bc

Carbon and nitrogen percentages in seedling tissues (*n* = 3). C:N ratio in plant tissues (*n* = 3). The soluble carbohydrate (SC), starch, and total phenolic compound concentrations (*n* = 6) in the seedling tissues were calculated for the needles, stems, and roots, respectively, and were normalized to the DW. Structural carbohydrates ratios (^a^) were estimated on the basis of FTIR spectra A1375 characteristic for cellulose and hemicelluloses and A1515, characteristic for lignin. For full FTIR spectra see Supplementary File S2. Values followed by different letters are significantly different from each other at *p* ≤ 0.05 according to the HSD least significant difference test, if possible

*DW* dry mass, *ns* (^b^) not significant, *NFH* natural forest habitat, *DFH* disturbed forest habitat, *IH* initial habitat (for description, please see M&M section)

Soluble nonstructural carbohydrates (SC) were most concentrated in the needles, with the highest values in IH, despite lower C (%) and pigment levels (Table 1). The lowest SC levels were observed in DFH and NFH B needles (Table 1). Roots from IH exhibited the highest SC levels, despite low C (%), while stems consistently showed lower SC concentrations than other organs (Table 1).

Starch levels were greatest in needles from IH and declined from DFH to NFH but were not significantly higher than in other organs (Table 1). Natural habitats had the lowest starch levels across all organs (Table 1). Phenolic compounds followed a similar trend, being most abundant in needles, with increases observed in DFH B and IH B. Root phenolic levels were lowest in NFH (Table 1).

Structural carbohydrates, analyzed via FTIR, varied across habitats. Stems in disturbed habitats (notably IH) exhibited the highest concentrations, while roots showed less variation (Supplementary File S2). Needles had the lowest lignin levels, with no significant differences among treatments, whereas stems in IH showed the highest holocellulose:lignin ratios (Table 1).

### Markers of oxidative stress and tolerance

**Fig. 7:**
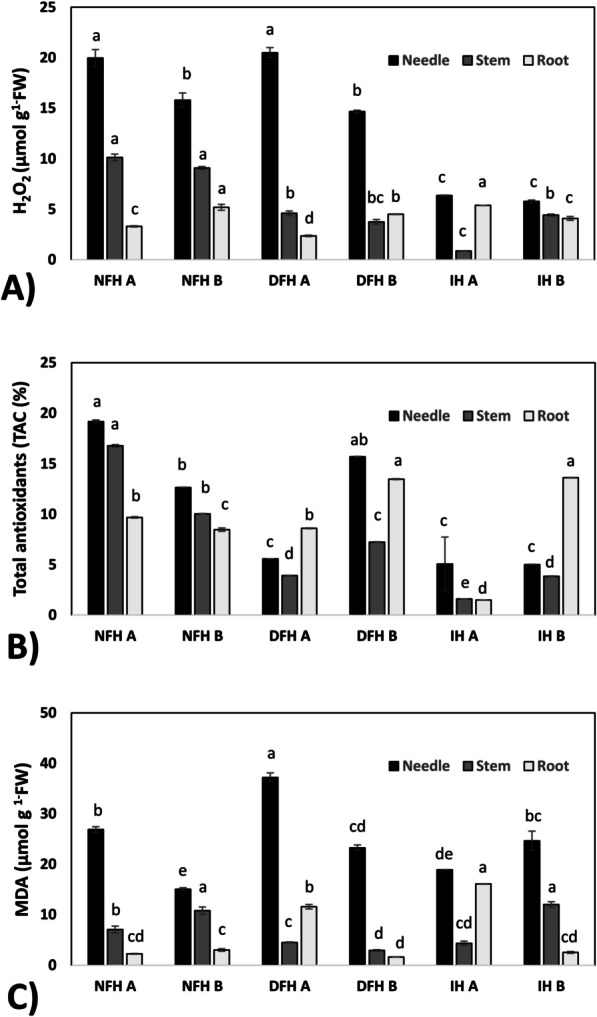
Oxidative stress markers. Levels of hydrogen peroxide (H_2_O_2_) (**A**), malondialdehyde (MDA) (**B**), and total antioxidant (**C**) concentrations in the needles, stems and roots of seedlings. Values are expressed as the means (*n* = 3) ± SEs. Different letters indicate significant differences (*p* ≤ 0.05) according to an HSD test. FW – fresh weight; NFH – natural forest habitat; DFH – disturbed forest habitat; IH – initial habitat (for description, please see M&M section)

The highest levels of reactive oxygen species (ROS) estimated on the basis of H_2_O_2_ concentration were consistently observed in the needles of the fir trees compared to those in the stems and roots (Fig. 7A). Notably, fir trees growing in NFH and DFH habitats exhibited the highest levels of H_2_O_2_ in their needles, while the lowest levels were detected in the needles of trees growing in IH (Fig. 7A). This trend was slightly more pronounced in the stems, where even some tendency toward decreasing H_2_O_2_ levels with increasing environmental pressure was observed (NFH → DFH → IF; Fig. 7A). In contrast, the levels of radicals in fir roots varied significantly between the analyzed variants, but no clear trends linking ROS levels with environmental pressure intensity were observed (Fig. 7A).

The total antioxidant capacity (TAC), represented as the percentage reduction in DPPH activity, exhibited significant variations among the different plant organs. In our study, the TAC (%) was associated with ROS concentration (Fig. 7A vs. 7B). Compared with those of the other organs studied, the TAC (%) was greater for the Fir needles. Remarkably, the roots displayed the lowest antioxidant activity (Fig. 7B). Furthermore, the NFH and DFH variants consistently demonstrated the highest TAC (%) in needles as well as stems (in which, in general, a decrease in TAC (%) was observed in the order NFH → DFH → IF, similar to what was detected for ROS). Interestingly, there was no significant difference in the antioxidant activity between the roots of the NFH and DFH variants. Conversely, the IH variant presented the lowest TAC (%) in the root samples (Fig. 7B).

MDA is widely utilized as a reliable indicator of the level of lipid (and generally macromolecule) damage occurring during oxidative stress in plants. The highest level of MDA was detected in the needles of fir trees compared to in the stems and roots (Fig. 7C). The level of oxidative changes remained consistently high in the needles of fir trees growing in all three habitats (NFH, DFH, and IH). In the case of stems and roots, the levels of the oxidative change indicator remained low in all three analyzed habitats, without any visible trend (decrease or increase; Fig. 7C).

## Discussion

### Conditions of fir seeds and the role of successful seed germination

The first step of spontaneous recolonization of abandoned quarry areas is associated with successful germination of seeds. Such success relies on available resources, i.e., soil nitrogen or carbon [8]. Soil microbial activity and chemical properties were significantly influenced by the disturbance regime, with DFH and IH conditions showing notable deviations from the optimal conditions observed in NFH. For silver fir, the abovementioned success most likely relies inter alia on the orthodox character of fir seeds [42, 43] and on seed viability and quality [44], all of which enable seeds to survive the most unfavorable periods until the optimal time for germination in the quarries (e.g., when rainwater is much more readily available). High interspecific variability resulting from genetic differences or phenotypic plasticity of seeds, as well as their high quality reflected in seed mass and energy storage [45], is considered a valuable source of acclimatization and adaptation of young seedlings to new climatic conditions [46]. Although we lack direct data on the physiological condition of the parent trees or the quality of their seeds, the observed success of natural regeneration in the quarry suggests that the seeds may have originated from relatively unstressed trees in the nearby fir forest. The EUFORGEN technical guidelines [47] note that *A. alba* has effective wind-dispersed seed propagation, enabling it to colonize disturbed sites, including abandoned pastures and pioneer pine stands. This could partly explain their high viability and early establishment capacity under the extremely harsh quarry conditions, as evidenced by the presence of numerous naturally regenerating seedlings.

### The impact of prolonged exposure to stressful conditions on metabolism adjustments of fir seedlings

The ability of silver fir seeds to germinate in harsh environments like IH does not fully explain the long-term survival of 2-year-old (and older) seedlings, as active quarries expose plants to continuous stressors such as drought, high-light conditions, nutrient deficiencies, and poor soil structure. In our study, these stress conditions were confirmed by field measurements, including 3–fivefold higher insolation levels in the quarry than in forest habitats (DIFN values), low organic matter and water-holding capacity, and shallow, stony soils with minimal humus development (Fig. 2 and 3). These conditions typically lead to overproduction of radicals, which may lead to activation of antioxidant defence systems but especially to oxidative damage like lipid peroxidation and accumulation of MDA as a secondary marker [48]. While oxidative stress is often marked by increased levels of ROS and MDA, we observed no such escalation in response to environmental gradients. Needles displayed the highest MDA levels compared to stems and roots, likely due to their direct exposure to stressors and high metabolic activity [40]. This accumulation of MDA in needles is consistent with the intense light exposure and nutrient scarcity in the quarry habitat, which likely enhance ROS generation and oxidative lipid damage in photosynthetically active tissues.

Changes in needle pigments, such as reduced chlorophyll and increased carotenoids, suggest adaptive mitigation to prevent photoinhibition and excess heat under high insolation [49]. Despite reduced photosynthetic pigments, SC levels were highest in IH needles, most probably due their role as osmoprotectants that shield plants from desiccation and high-light stress [48, 50], indicating nevertheless sufficient efficiency of the entire photosynthetic process. Enhanced phenolic compounds in IH needles, known antioxidants, further contributed to stress mitigation [21, 50]. These responses are closely linked to environmental pressures specific to IH, such as intense solar radiation and limited soil nutrients. These mechanisms likely explain why ROS and TAC levels did not escalate, even under IH conditions.

The elevated SC concentrations in needles were reflected in stems, although roots from IH showed reduced C (%). The initial nature of the ground, reflected by the lower soil LOI and WHC in IH likely resulted in diminished ECM fungal biomass, reducing fungi-induced C flux into roots [7, 51]. Despite this, ECM colonization was complete in all seedlings, underscoring the importance of these symbiotic relationships for survival in harsh environments [14, 15]. Ectomycorrhizal fungi facilitated efficient nutrient uptake, particularly nitrogen, from barren soils, which is crucial for silver fir growth in quarries [13].

The presence of diverse ECM fungal species in NFH and reduced diversity in IH highlight the adaptability of silver fir seedlings to varying conditions. Ectomycorrhizal associations not only support nutrient uptake but also enhance resilience against environmental stresses, enabling silver fir recolonization in nutrient-poor quarry soils [8, 52]. These findings suggest that silver fir seedlings rely on metabolic adjustments and ECM symbioses for survival and growth under prolonged environmental pressures in disturbed habitats.

Nevertheless, we acknowledge that the lack of classical growth-related metrics, such as fresh weight or root length, limits the possibility of fully integrating physiological outcomes with morphological performance. While our study prioritized sensitive biochemical indicators to reveal early acclimatization responses under field conditions, future research should incorporate these morphological traits to provide a more comprehensive picture of how silver fir seedlings balance metabolic adaptation with structural development in disturbed environments. This approach would also help verify whether the observed biochemical stability translates into enhanced biomass accumulation or growth efficiency across contrasting habitats.

Interestingly, the tallest fir seedlings were found at the quarry edge (IH B), while the shortest were located at the quarry bottom (IH A). These contrasting growth responses within a single habitat type likely reflect fine-scale microhabitat variability, including slope exposure, soil properties, and water availability. The edge zone (IH B) may offer slightly improved conditions, such as better soil aeration, higher organic matter content, or reduced compaction, while seedlings from the quarry bottom (IH A) likely face harsher conditions, including rapid surface drying, physical disturbance, or mechanical stress from substrate instability. Additionally, seedlings from IH A had the lowest C content in roots, which may signal weaker belowground biomass development and less efficient carbon allocation. These findings support the conclusion that even within broadly defined habitat types, local heterogeneity strongly influences seedling establishment and plant performance [6].

### Metabolic changes indicative of acclimatization and high plasticity in fir seedlings

ECM is considered as a major tolerance mechanism in plants [13] explaining in part the good condition of seedlings growing in disturbed habitats. Nonetheless, in the analyzed silver fir seedlings, we not only did not observe symptoms of severe cellular stress, but some of the observed metabolic modifications were most likely adaptive (or rather acclimatizing) in nature.

One of the most important metabolic modifications was an increase in the relative abundance of protective carotenoids [53]. An increase in carotenoids is known to increase plant tolerance to abiotic stresses [21, 54], as this group of foliar pigments is the most efficient quencher of ^1^O_2_ [55]. The presence of carotenoids may be attributed to the high efficiency of needle protection against radical-induced high light in extremely exposed IH habitats.

In addition to leaf pigments, starch concentrations are one of the few analyzed biochemical features that are clearly related (in needles) to environmental pressure. Starch, as the most important foliar stored substance, enables quick remobilization, energy release and derived metabolites to help mitigate stress conditions when necessary [56]. Importantly, such a reserve would not adversely affect tree growth [57]. High starch (and SC) concentrations in needles likely contributed to carbon retention in the aboveground parts of young firs in disturbed habitats, resulting in the previously mentioned reduced root C (%).

On the other hand, under highly exposed IH, we expected that the epicuticular wax layer would exhibit several morphological traits that could be interpreted as modifications with protective functions against, e.g., strong radiation and water loss [58]. However, neither the general characteristics of the stomata (data not shown) nor the morphology of the epicuticular waxes differed significantly between the analyzed variants.

In contrast, some acclimatizing mechanistic adjustments, namely, more structural cell wall carbohydrates, were found in fir stems but not in roots, indicating that the seedlings growing in the quarry were strengthened [59]. The lack of clear strengthening in IH roots was somewhat surprising, as increases in root structural carbohydrates are usually considered beneficial features for roots growing in compacted soils [60]. Still, this could be related to the observation that in stony soil, elongated roots exhibit reduced rigidity, which is attributable to the heightened cell wall relaxation necessary for generating the increased growth pressure needed in strong soil conditions compared to in weaker ones [61].

Despite prolonged abiotic stress in IH, ROS and MDA levels in fir seedlings remained within moderate ranges, as seen in other conifers under stress [19], suggesting the absence of critical oxidative damage. Rather than systemic antioxidant activation, seedlings showed organ-specific responses, notably increased carotenoids and phenolics in needles—known non-enzymatic antioxidants that support localized redox control [62]. This restrained antioxidant profile aligns with drought-adapted strategies observed in fir populations from dry habitats [63]. Our findings thus confirm that seedlings developed specific biochemical acclimatizations, supporting hypothesis (i), via subtle, targeted responses rather than broad antioxidant upregulation. These patterns echo results by Kijowska-Oberc et al. [64], showing that early-stage redox balance can be maintained without strong stress signalling.

## Conclusion

In conclusion, the absence of pronounced biochemical signals indicating severe stress or compromised seedling growth, combined with observed metabolic shifts and adaptive modifications, suggests that *A. alba* seedlings thriving in quarry habitats are not merely tolerating stress but actively acclimatizing to harsh conditions from the earliest growth stages. Despite prolonged exposure to extreme environmental pressures, including nutrient-poor soils, high insolation, and water limitations, the seedlings exhibited stable ROS and antioxidant levels, enhanced structural reinforcement (increased lignin), protective foliar pigment shifts (higher carotenoid and xanthophyll contents), and efficient carbohydrate storage. These metabolic adjustments indicate a strong capacity for physiological plasticity, which supports their survival and growth in disturbed environments.

Furthermore, full mycorrhizal colonization, even in the most degraded sites, highlights the crucial role of symbiotic fungi in nutrient acquisition and stress mitigation, reinforcing the resilience of silver fir in extreme conditions. Our study confirms the mitigation of the negative effects of prolonged stress exposure, supporting the hypothesis that plants continuously exposed to challenging conditions develop stabilizing mechanisms that enhance their long-term survival.

There are relatively few biochemical studies focusing on naturally regenerating climax species, particularly under in situ conditions of early-successional, environmentally extreme habitats such as active quarries. While some previous studies — including from Poland (e.g. [65]) — have investigated the growth and adaptation of young *A. alba* individuals in various environments, reports specifically addressing their biochemical plasticity and stress physiology in such disturbed settings remain limited. Our results provide novel insight into this gap, emphasizing the surprisingly high acclimatization potential of *A. alba* during early colonization stages. These findings challenge the long-held perception of silver fir as a strictly late-successional species dependent on stable forest conditions and suggest that it may play a more active and earlier role in forest succession on degraded lands than previously assumed [47]. This highlights its potential significance in ecological restoration and adaptive forest management strategies under ongoing climate and land-use change. Finally, our results open new directions for future research. Our data suggest that not only two-year-old seedlings but also older individuals can persist in quarry environments (Supplementary File S1). Future studies should assess whether and to what extent chronic heat, drought, or nutrient stress impacts the later stages of silver fir development — particularly in terms of biomass accumulation, structural traits, and long-term performance under such extreme conditions.

## Supplementary Information

Supplementary Material 1. Analyzed habitats and experimental sites - detailed data.

Supplementary Material 2. Structural carbohydrates - FTIR results

## Data Availability

The main data generated or analysed during this study are included in this published article and its supplementary information file. Additional datasets used and/or analysed during this study are available from the corresponding author upon reasonable request. The plant samples used in this study are maintained by the Institute of Dendrology Polish Academy of Sciences. The collection is housed at Parkowa 5, PL62035 Kórnik, Poland.
