# Supplementary material for: The high adaptive potential of Abies alba Mill. seedlings – biochemical and physiological studies of succession along the environmental gradient of a Cambrian quarry
Source: BMC Plant Biol. 2025 Jul 2;25:820. doi: 10.1186/s12870-025-06792-4 (PMC12220255; doi:10.1186/s12870-025-06792-4)

The high adaptive potential of *Abies alba* Mill. seedlings – biochemical and physiological studies of succession along the environmental gradient of a Cambrian quarry

Agnieszka Szuba, Ewelina Ratajczak, Tomasz Leski, Dominik Tomaszewski, Izabela Ratajczak, Gabriela Woźniak and Andrzej M. Jagodziński

Supplementary File S1

Analyzed habitats and experimental sites - detailed data

(**A**) Representative images of the forest floor (or its equivalent in the IH type habitats) showing the abundance of *Abies alba* regeneration and adjacent vegetation. From these habitats, 2-year-old *A. alba* seedlings were selected for further study. Scale: 20 cm

(**B**) Representative images of older (but still juvenile) *Abies alba* specimens occurring in the analyzed study plots (indicating the possibility of survival and further growth of the analyzed 2-year-old fir seedlings in all analyzed habitats), marked with a red arrow. Scale: 20 cm

(**C**) Representative images of the regenerative potential of secondary succession on an active quarry (IH habitats). Numerous vegetation is visible (with a predominance of common birch). In the immediate vicinity of the active quarry excavation (IH), a mature fir forest is visible, including the NFH (which is a bank of fir seeds in the analyzed experimental plots; marked with a red asterisk). Visible truck generating dust on the IH B experimental plot. Scale: 1m.

NFH – natural forest habitat; DFH – disturbed forest habitat, IH – initial habitat; A and B: experimental sites represented a particular habitat corresponding to the variants in Fig. 1 - 9. For IH: A: the bottom of the active quarry excavation; B: edge of active quarry excavation. For detailed description please see M&M section in the main manuscript.

The study area and the sample plots are related to their specific Cambrian sediments and its petrographic, mineralogical composition. The Upper Cambrian siliciclastic rocks of the Wiśniówka Massif (south-central Poland) are characterized by the ubiquitous pyrite that makes up, the two mineralization zones originated during the Late Cambrian. The fine material of the Cambrian pyrite are causing the acidity of the drainage water (Molenda et al., 2020). The oldest, Cambrian petrographic zone is composed of sedimentary intraclast-supported pyrite cement. Part of the pyrite consists of the framboidal pyrite that typically includes many arsenic-rich minerals (Szuba et al. 2023).

In the process of soil formation, the Cambrian bedrock has facilitated the development of a specific forest association known as *Abietetum polonicum* (EUR-28: Holy Cross fir forests - *Abietetum polonicum*; EUNIS: G3.134). Natural patches of this forest type are limited to a few locations with specific geology in this part of Poland. The tree species composition of these forests is characterized by the dominance of *Abies alba*, accompanied by *Pinus sylvestris* and *Picea abies*. The old *Abietetum polonicum* forest ecosystems feature a dense organic matter layer (NFHs). However, during the quarrying activity, the original *Abietetum polonicum* forest patches were removed, and the bedrock became available for obtaining hard Cambrian sandstone for construction purposes. The older quarry sites have undergone spontaneous succession. The 60-year-old areas (DFHs) are now covered by spontaneously developed *Pinus sylvestris* as the dominant species, along with *Betula pendula* and *Populus tremula* in the tree layer. The herb layer in these patches shows an outstanding abundance of *Lycopodium clavatum*, *Pyrola rotundifolia*, and *Ortilia secunda*. No soil layer has developed in these patches, and the rocks of different textures serve as the substrate. The sites under recent excavation (IHs) are mainly colonized by seedlings of *Abies alba* juv., *Betula pendula* juv., *Populus tremula* juv., *Pinus sylvestris* juv., and *Picea abies* juv. Surprisingly, there are no therophytes present as the first colonizers. Among the herbaceous plants, the scarce individuals of *Tussilago farfara* and *Calamagrostis epigejos* are the most abundant. In the wettest parts of the recently excavated sites, *Phragmites australis* occurs (see also: Fig. 1).

The studied quarry represents a less common type, specifically a Cambrian sandstone quarry. These are among the oldest geological formations exposed at the surface in this region and are characterized by extremely poor, mineral-rich substrates, very low organic matter, and limited water retention. These conditions create harsh environments, quite unlike those found in more typical limestone, basalt, or granite quarries. Regarding succession:

- In the freshly excavated part of the quarry (IH), where mining has only recently ended, we observed signs of primary succession. Vegetation is just beginning to establish on bare mineral ground, with only single individuals of pioneer species. Interestingly, despite these extreme conditions, *Abies alba* seedlings are already present, which is notable given the species' usual association with mature, stable forests.
- In the older quarry areas (DFH), where extraction ended about 60 years ago, the vegetation is more developed. Here, we observed patches of coniferous forest and the presence of *A. alba* seedlings in both the herb and shrub layers, which indicates a more advanced stage of colonization—though the site remains relatively young in ecological terms.

Because Cambrian sandstone outcrops are rare in temperate Europe, direct comparisons with similar quarries are difficult. Most ecological studies on quarry succession in the region have been conducted on limestone or basalt, which differ significantly in soil chemistry and fertility. This makes our site ecologically unique, and potentially valuable for studying forest regeneration and species plasticity under nutrient-poor, oligotrophic conditions.

Molenda, T., Ciupa, T., Suligowski,R. 2020. The properties of reservoir water in post-mining excavations of Cambrian and Devonian quartzite sandstones (Holy Cross Mountains). Environ. Earth Sci. 79, 310

Szuba, A., Ratajczak, E., Leski, T., Jasińska, A.K., Hanć, A., Piechalak, A., Woźniak, G., Jagodziński, A.M. 2023. Physiological response of adult *Salix aurita* in wetland vegetation affected by flooding with As-rich fine pyrite particles, Sci. Total Environ. 865: 161197

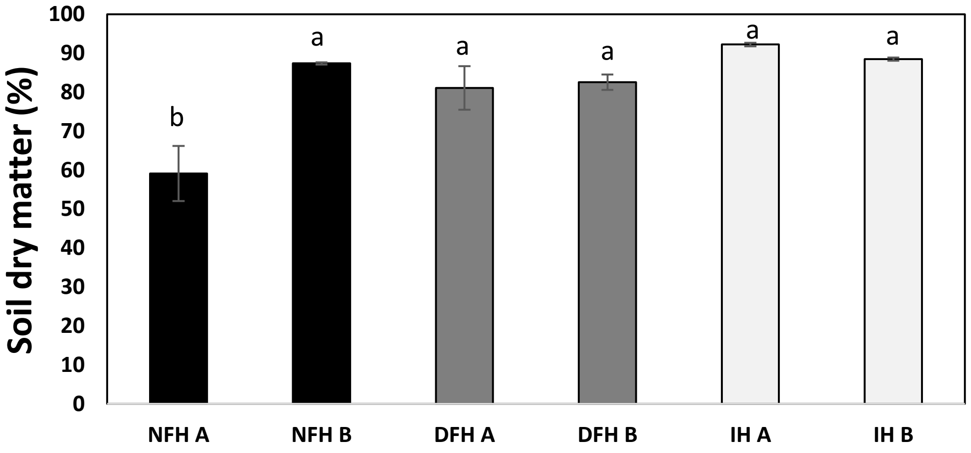

Soil dry matter percentage was calculated for particular plots. NFH – natural forest habitat; DFH – disturbed forest habitat; IH – initial habitat (for description, please see M&M section). Different letters indicate significant differences according to the HSD post hoc test (*n=5*).

(A)
NFH A

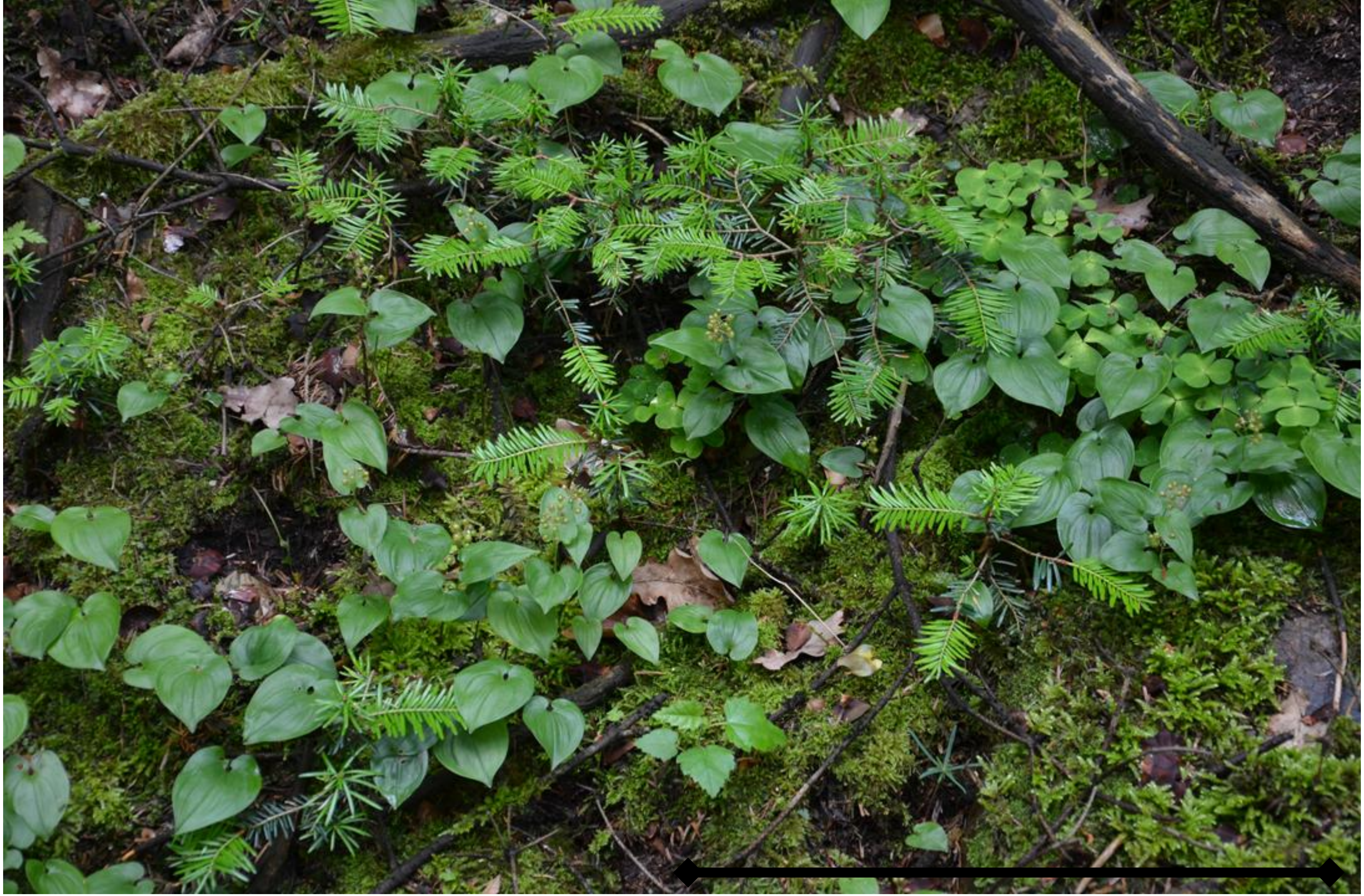

(**A**)
NFH B

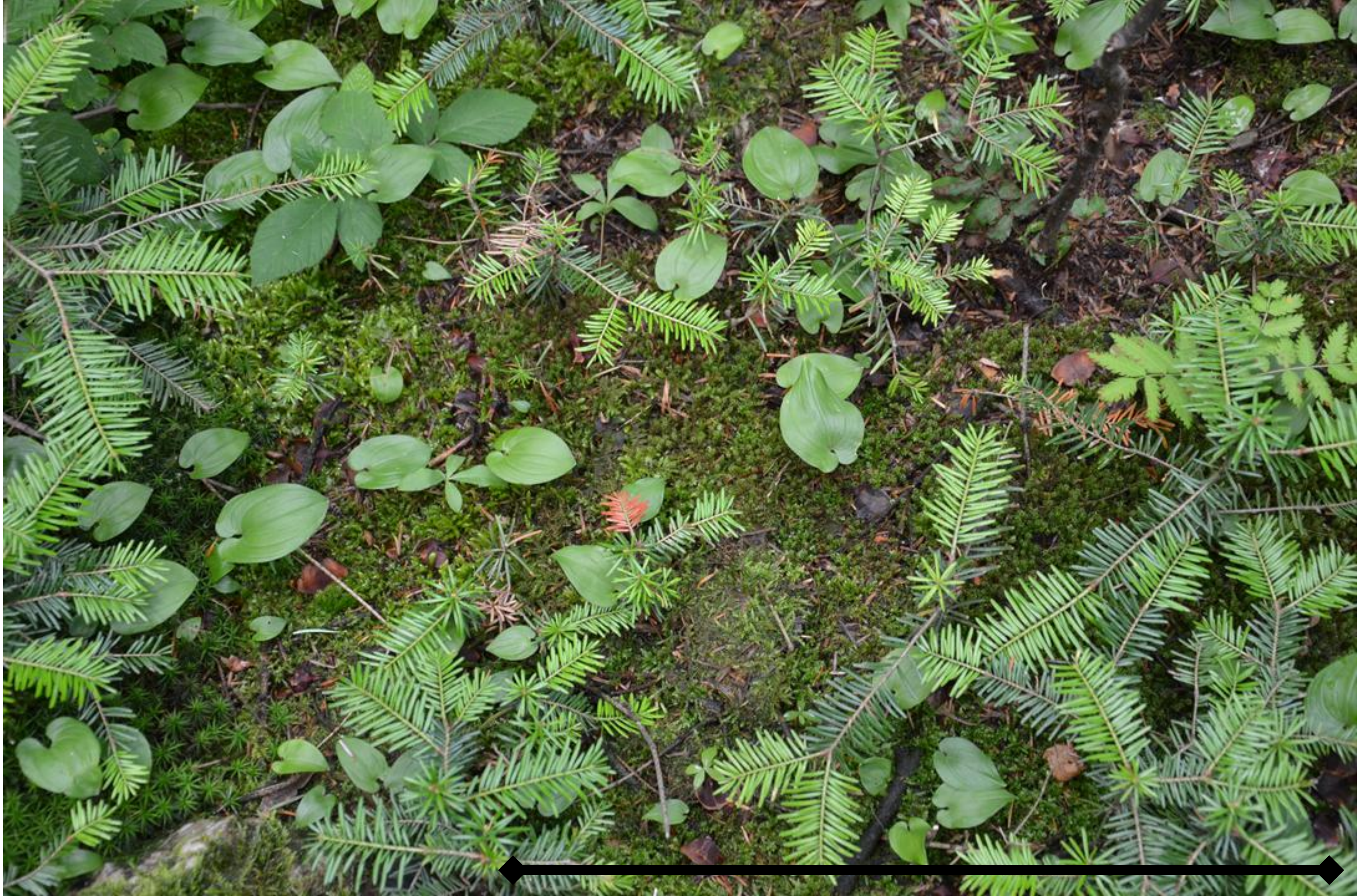

(**A**)
DFH A

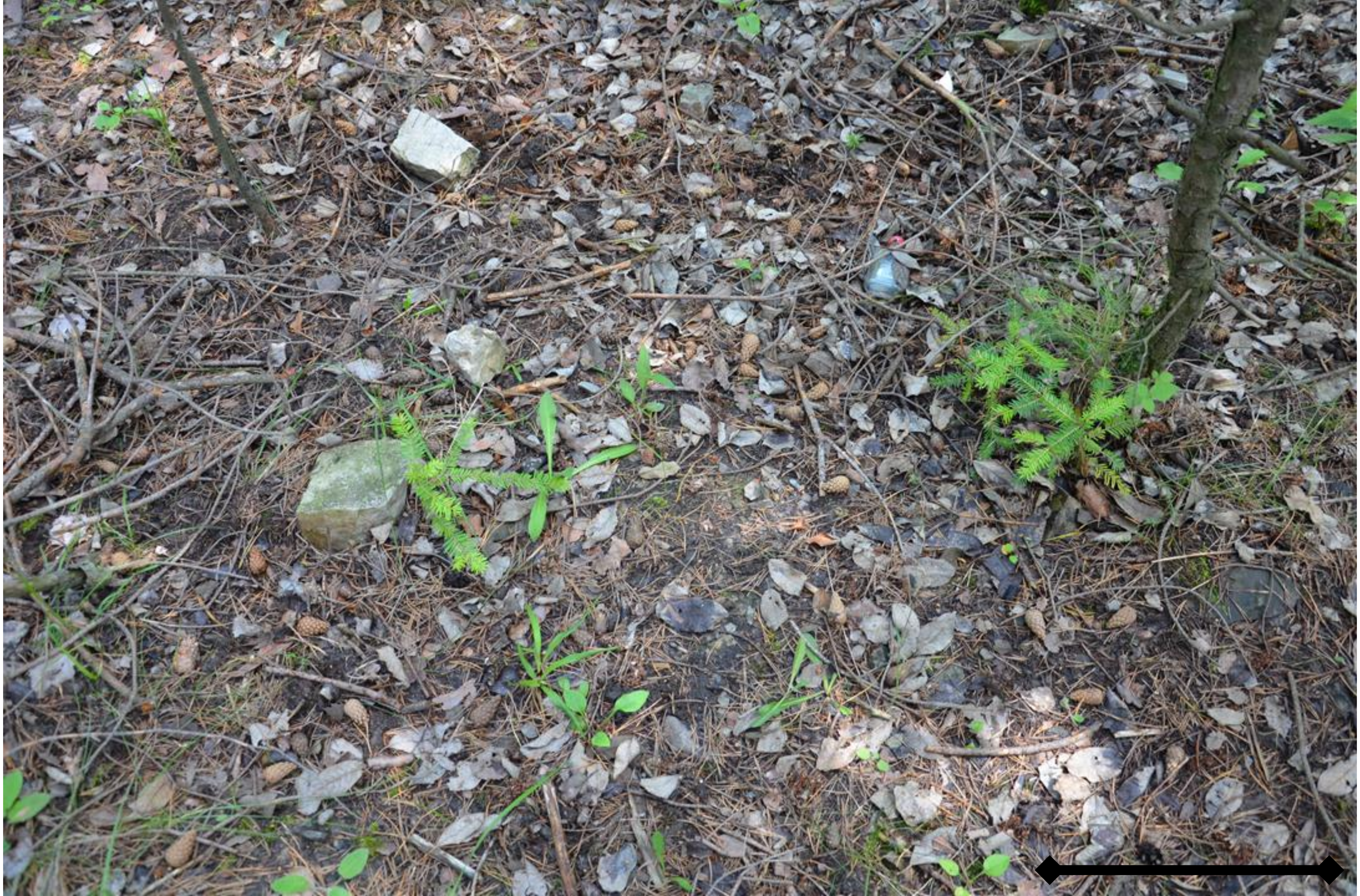

(**A**)
DFH B

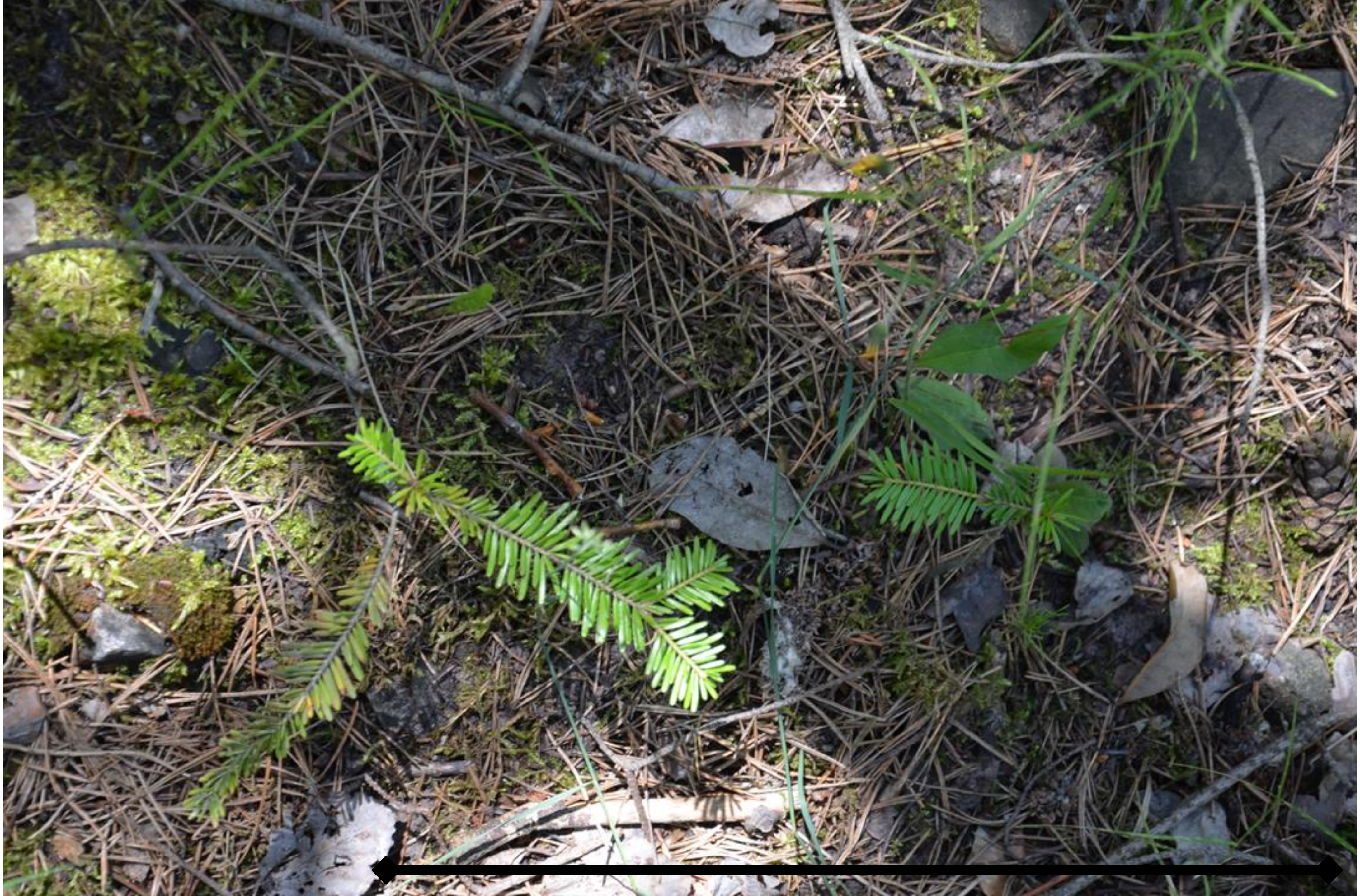

(A)
IH A

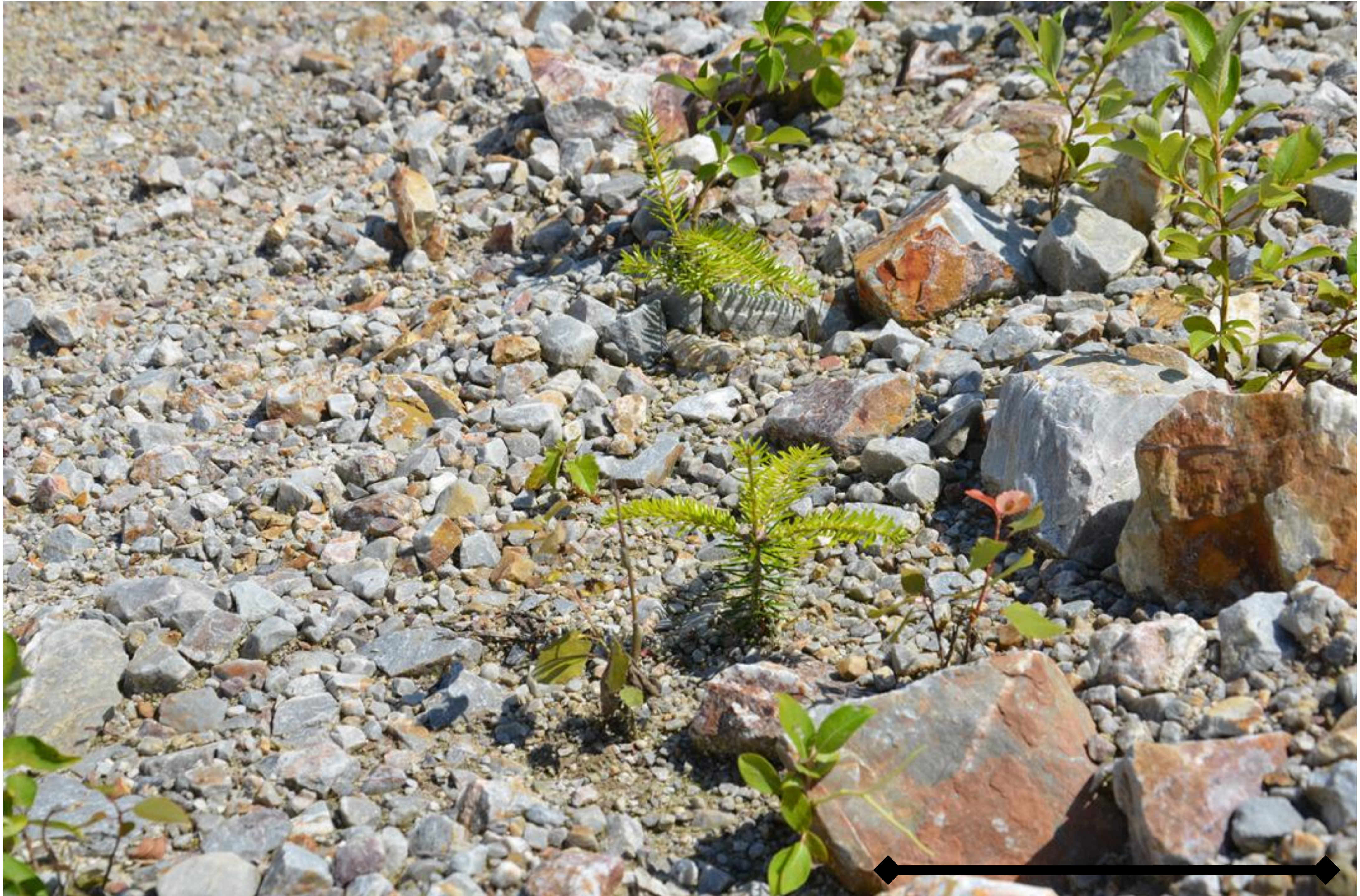

(**A**)
IH B

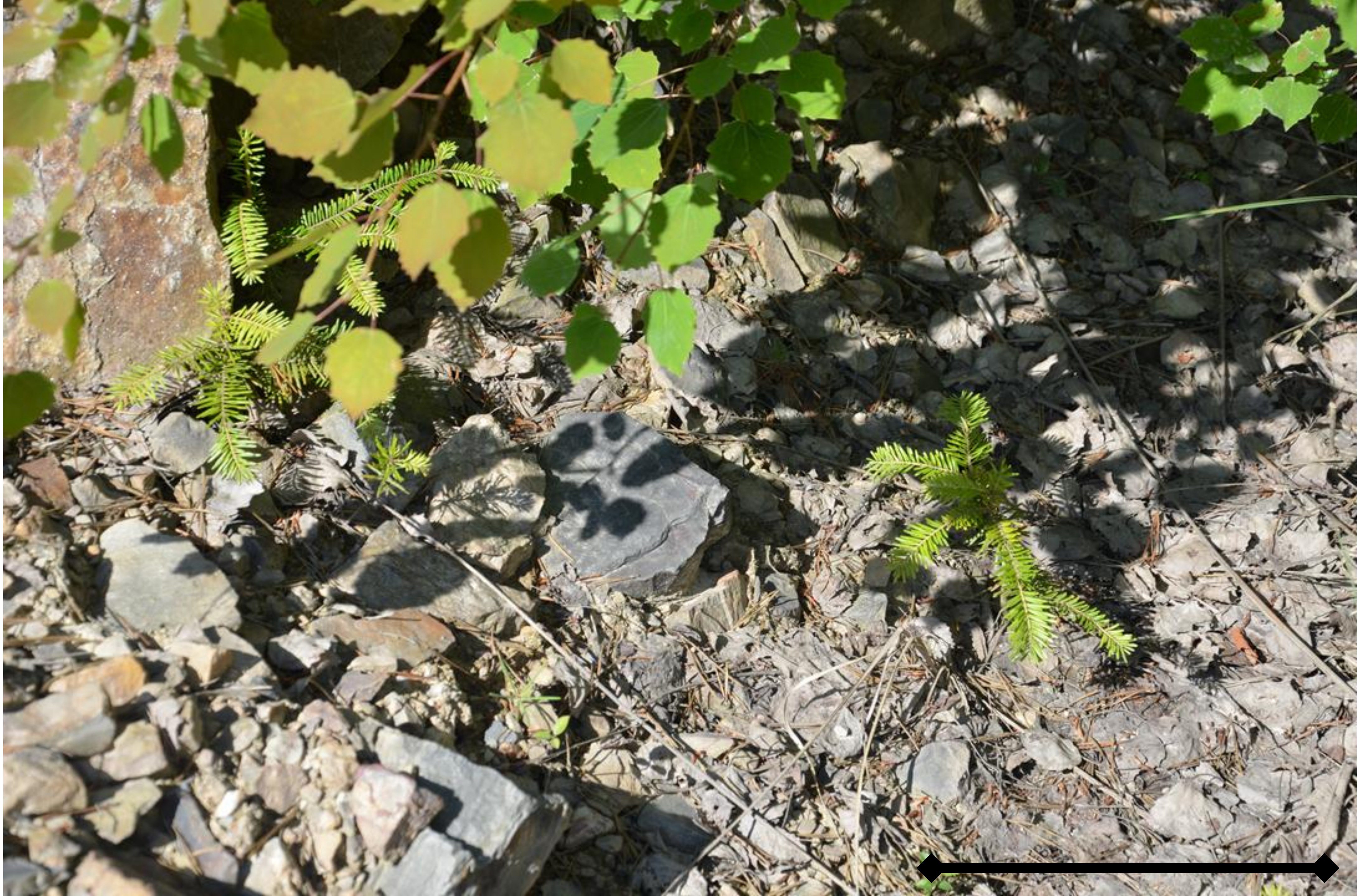

(B)
NFH A

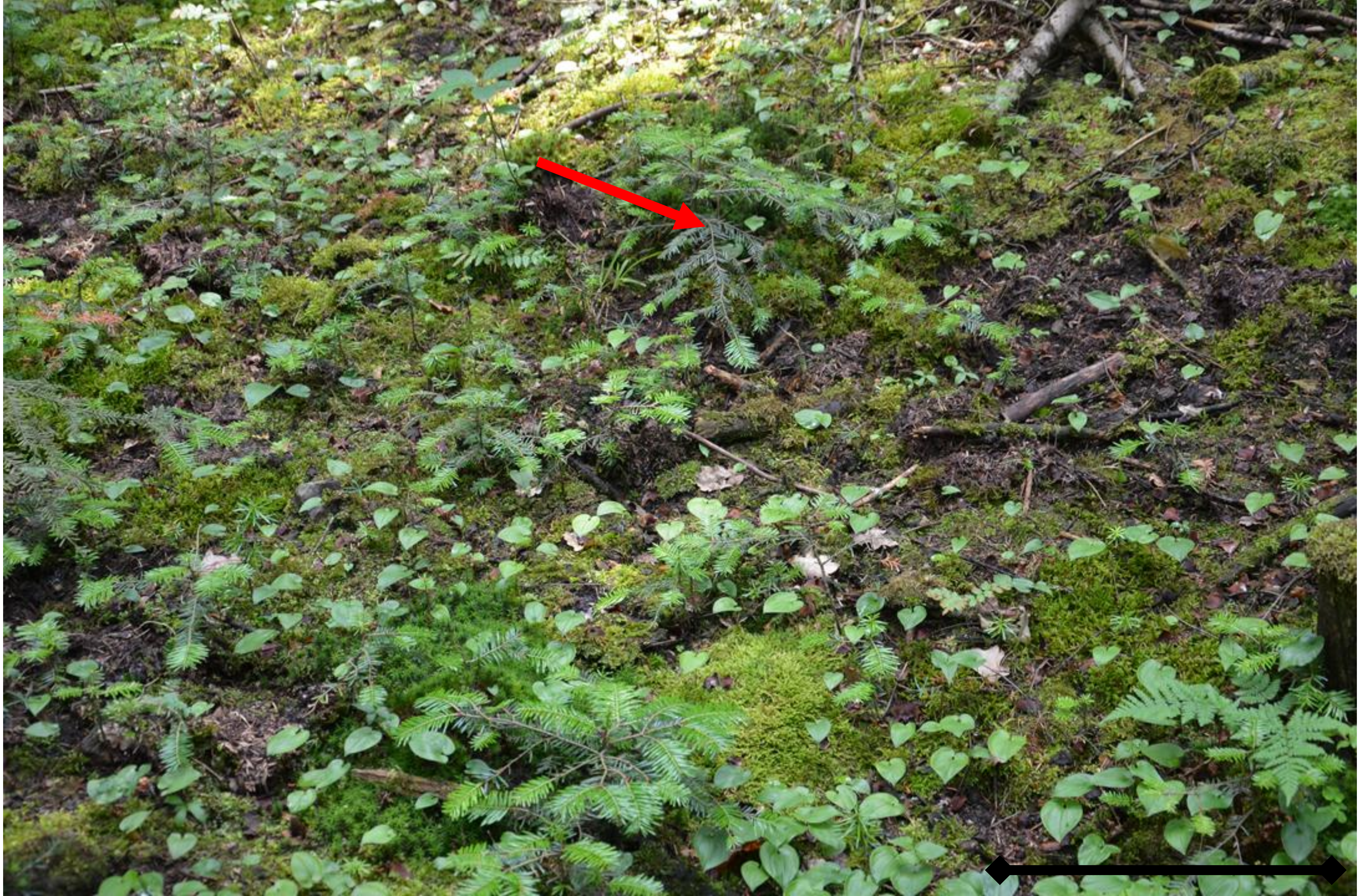

(B)
NFH B

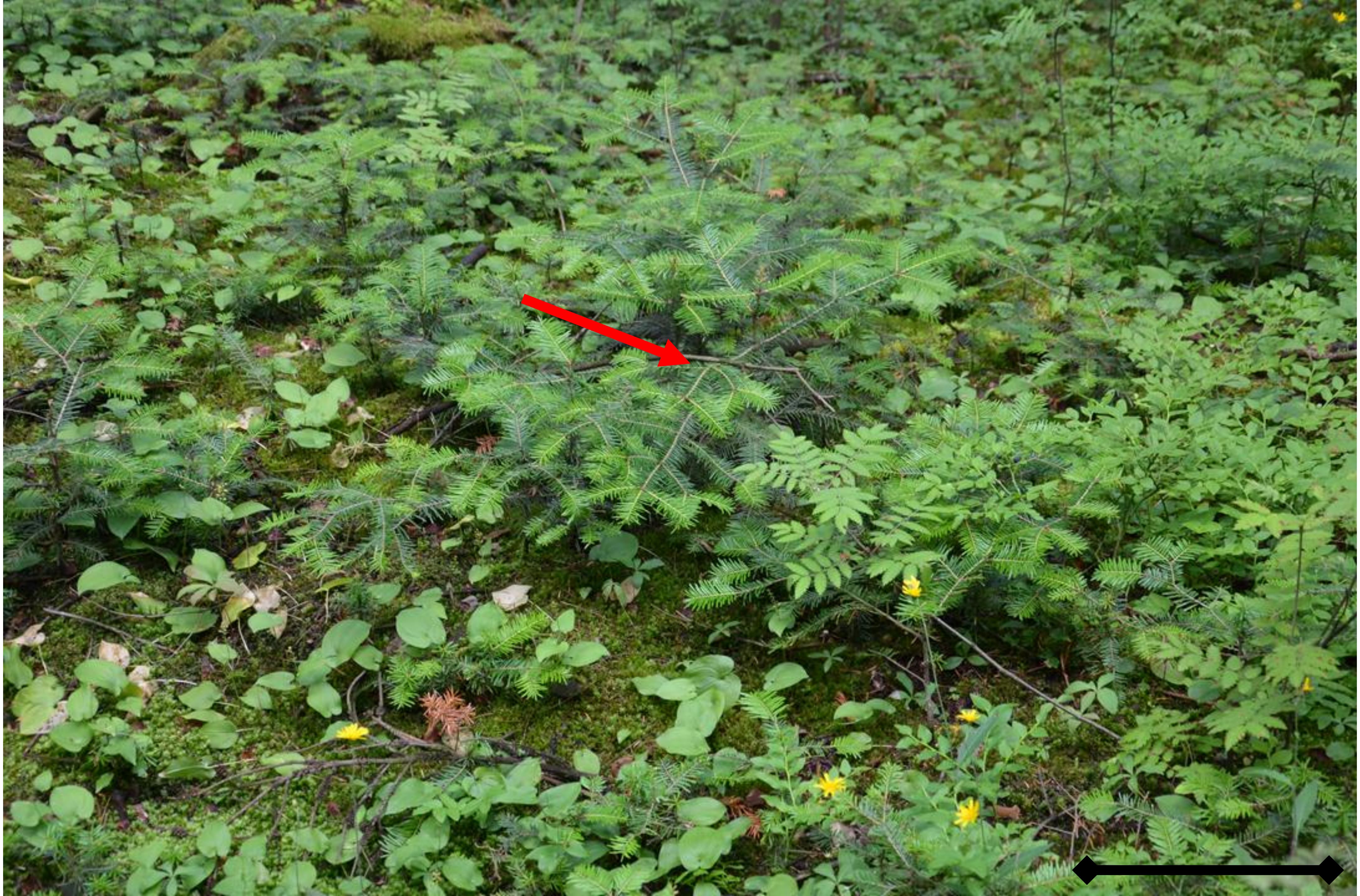

(**B**)
DFH A

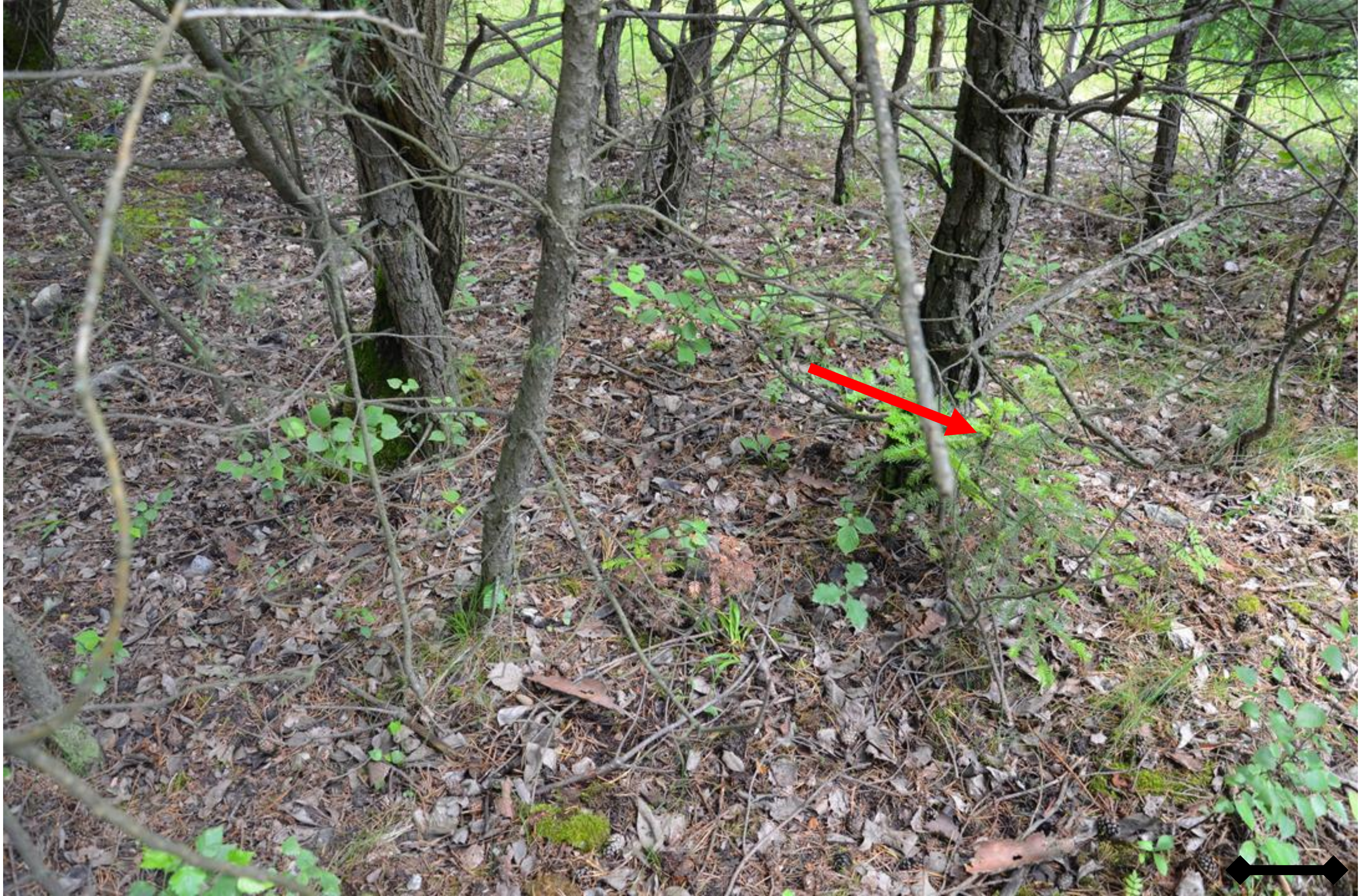

(B)
DFH B

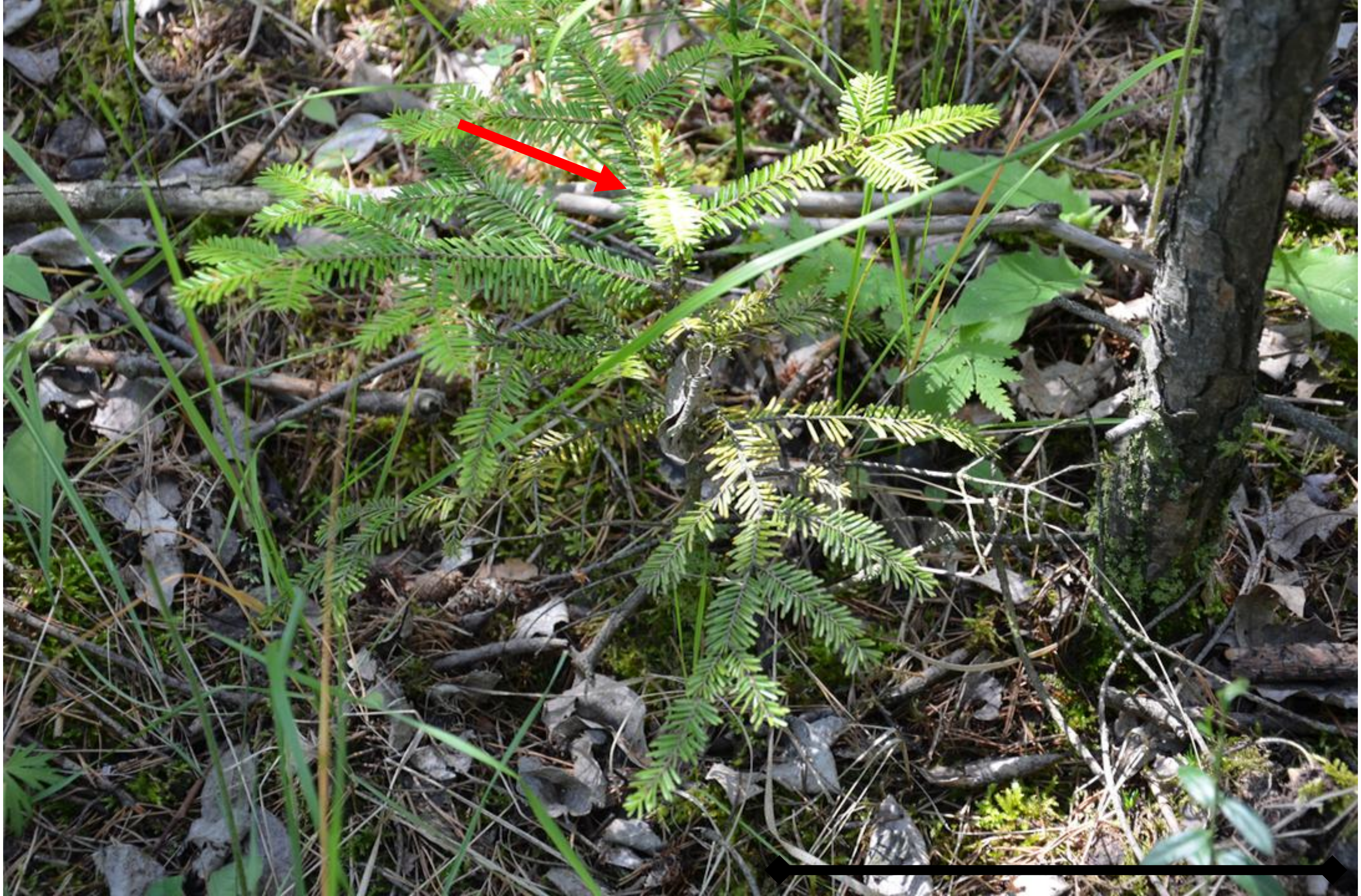

(B)
IH A

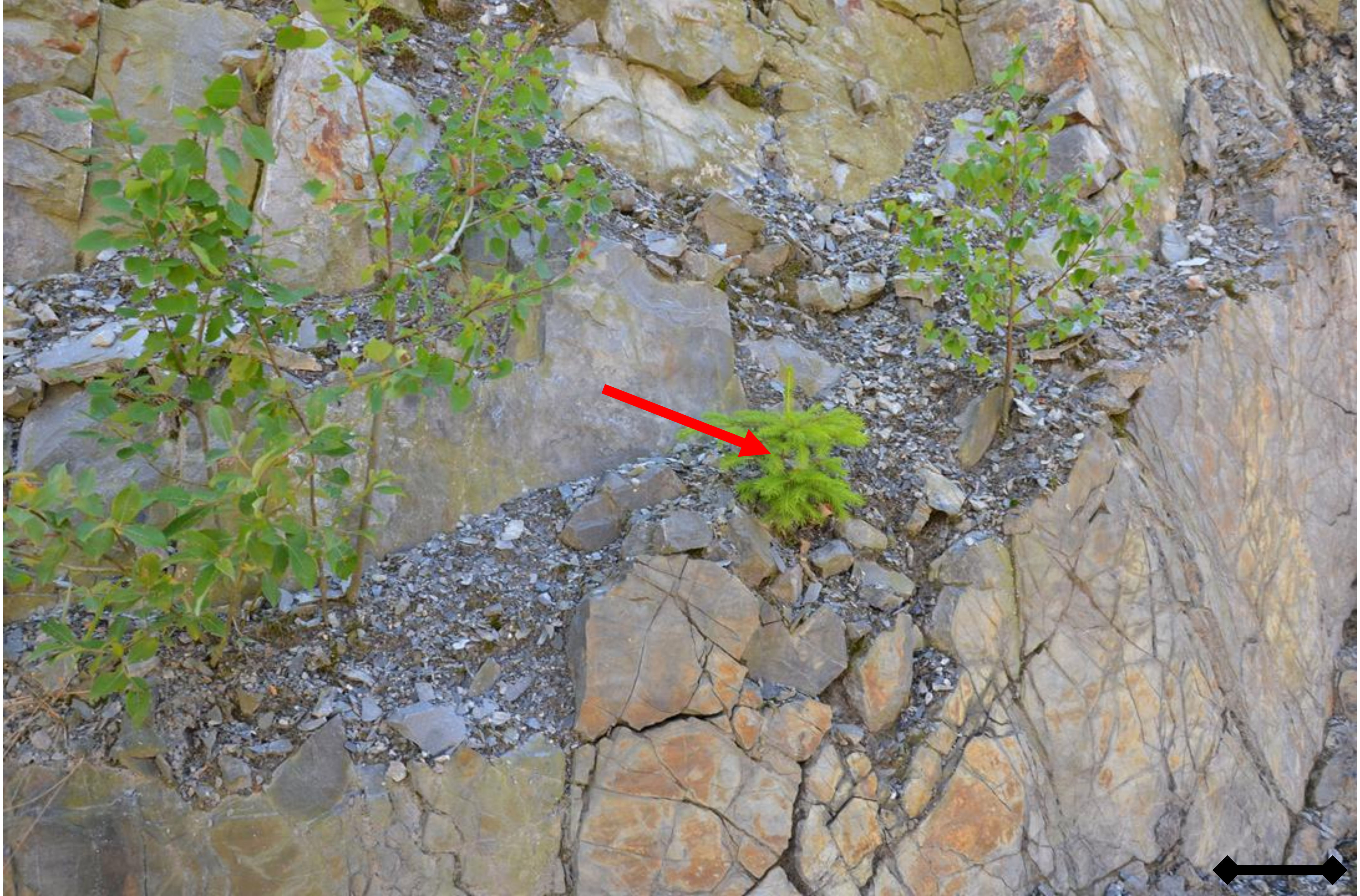

(B)
IH B

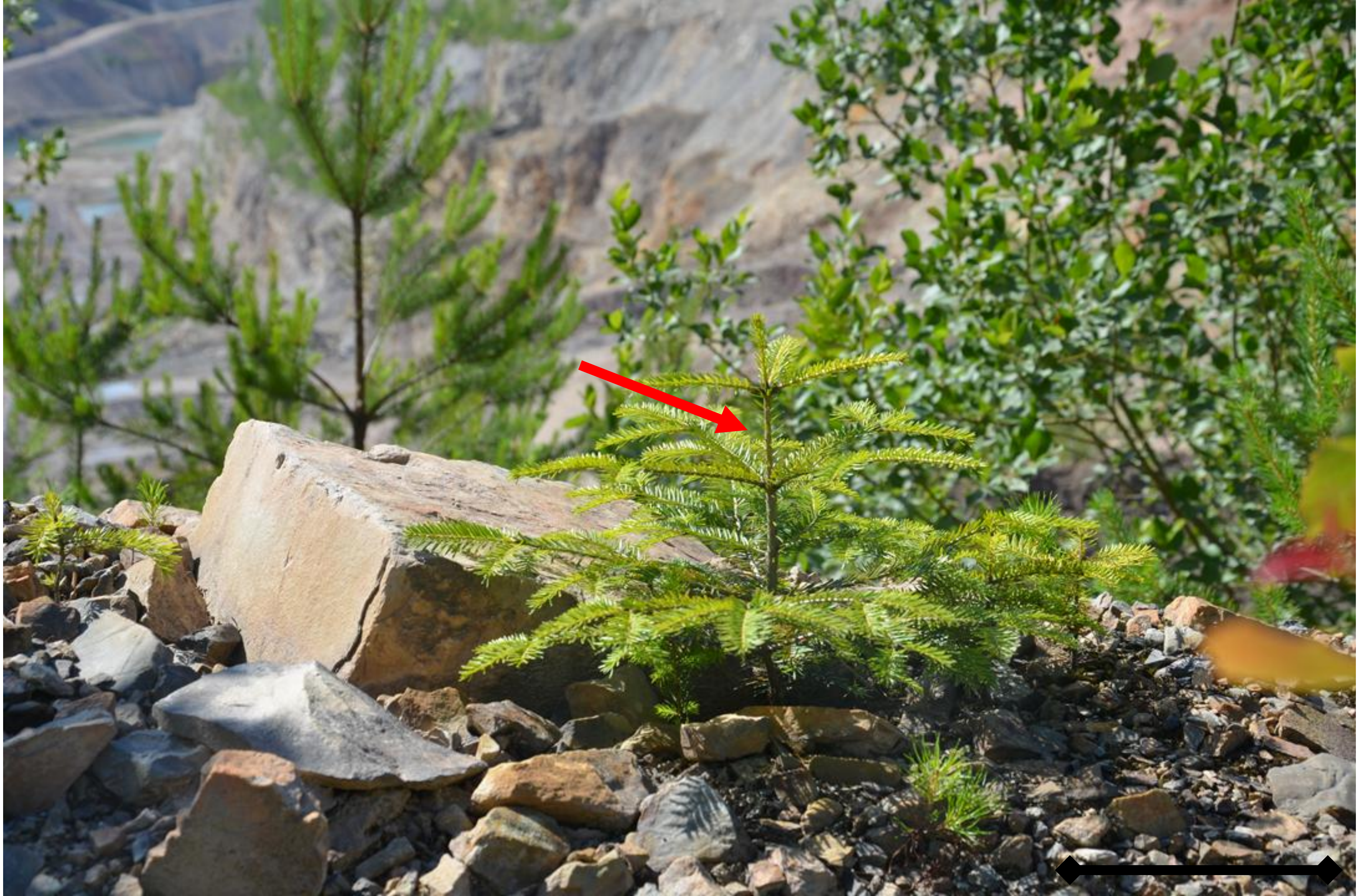

(**C**)
NFH A/B

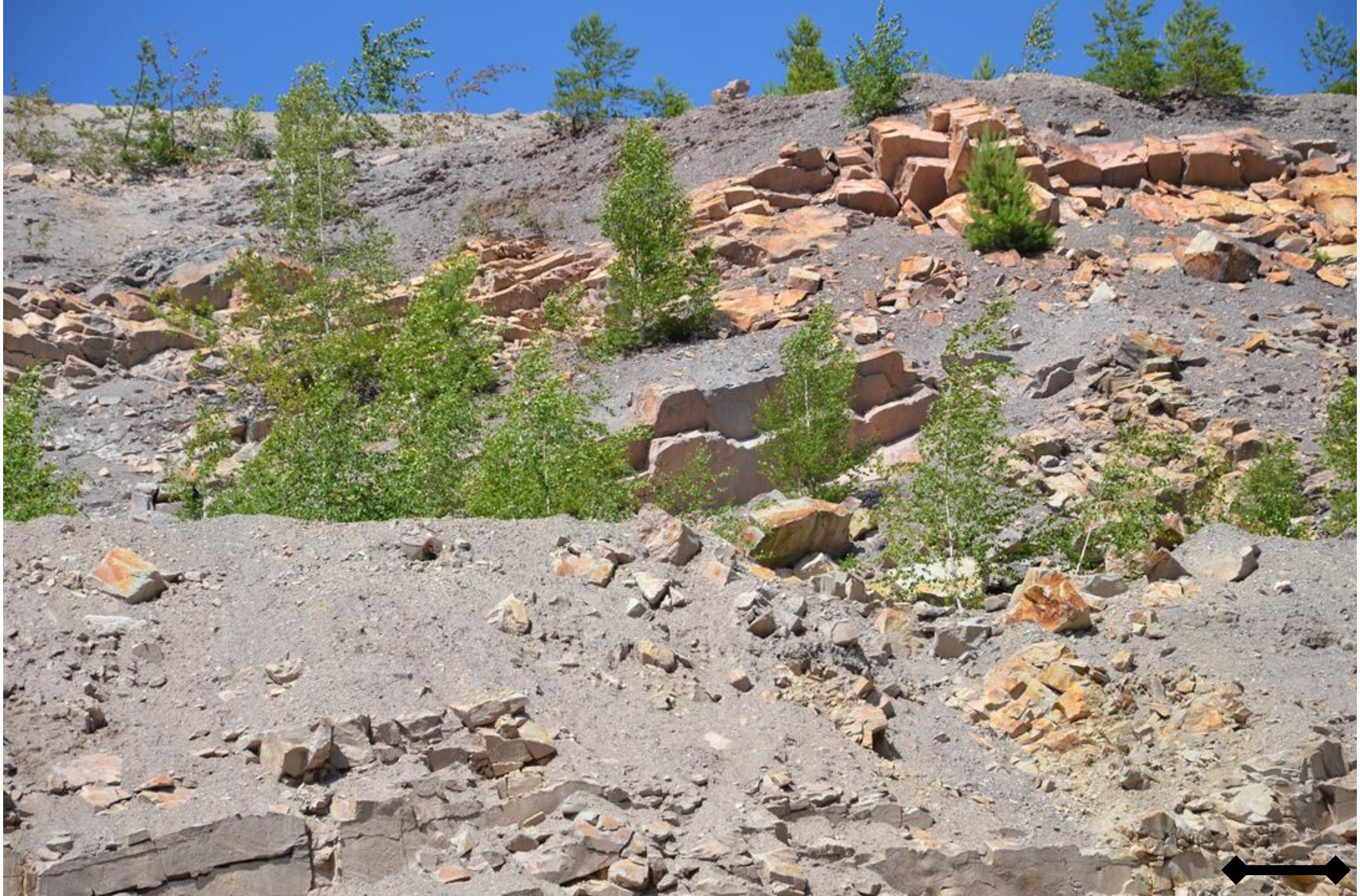

(**C**)
NFH A/B

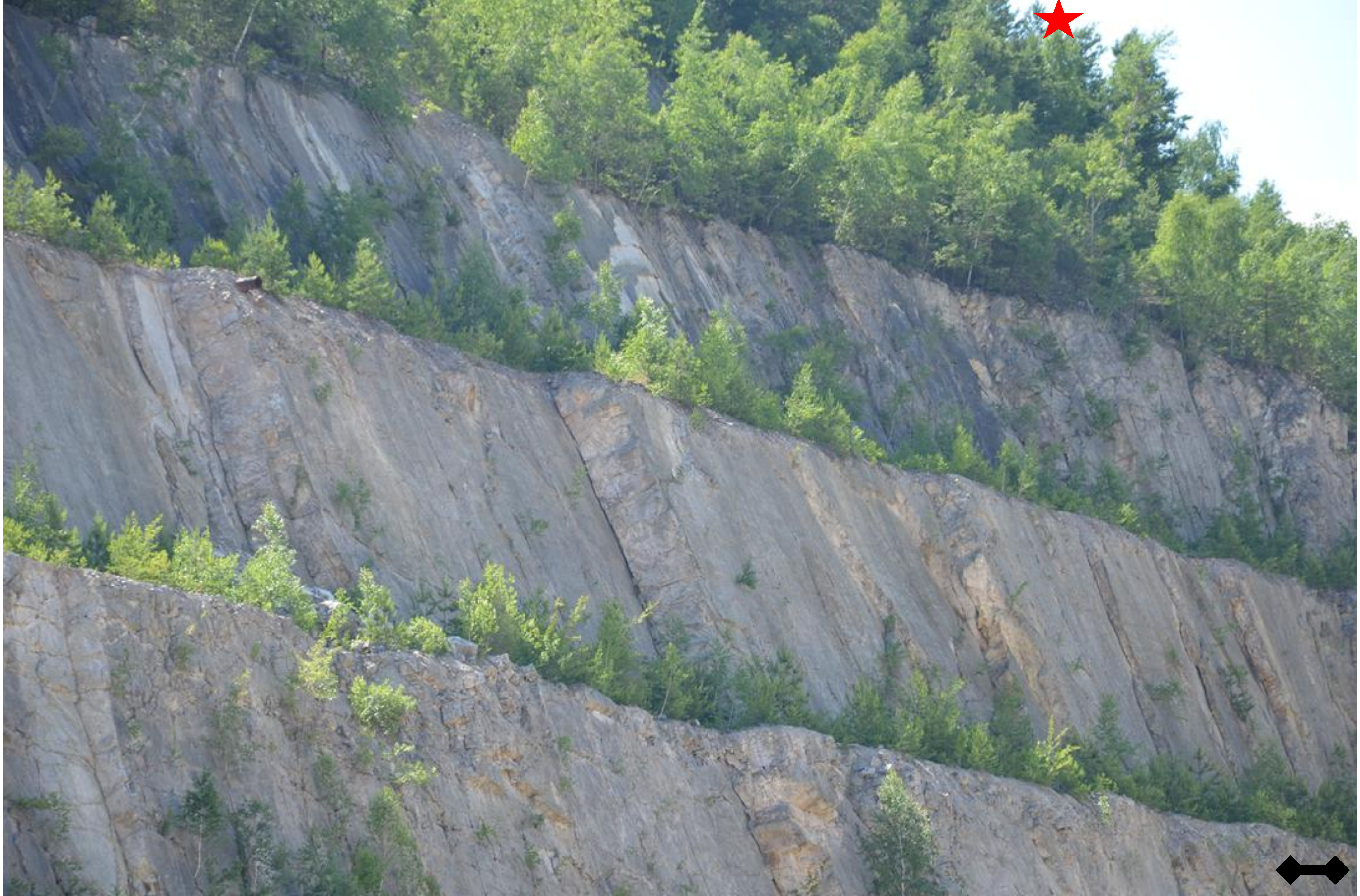

(C)
NFH B

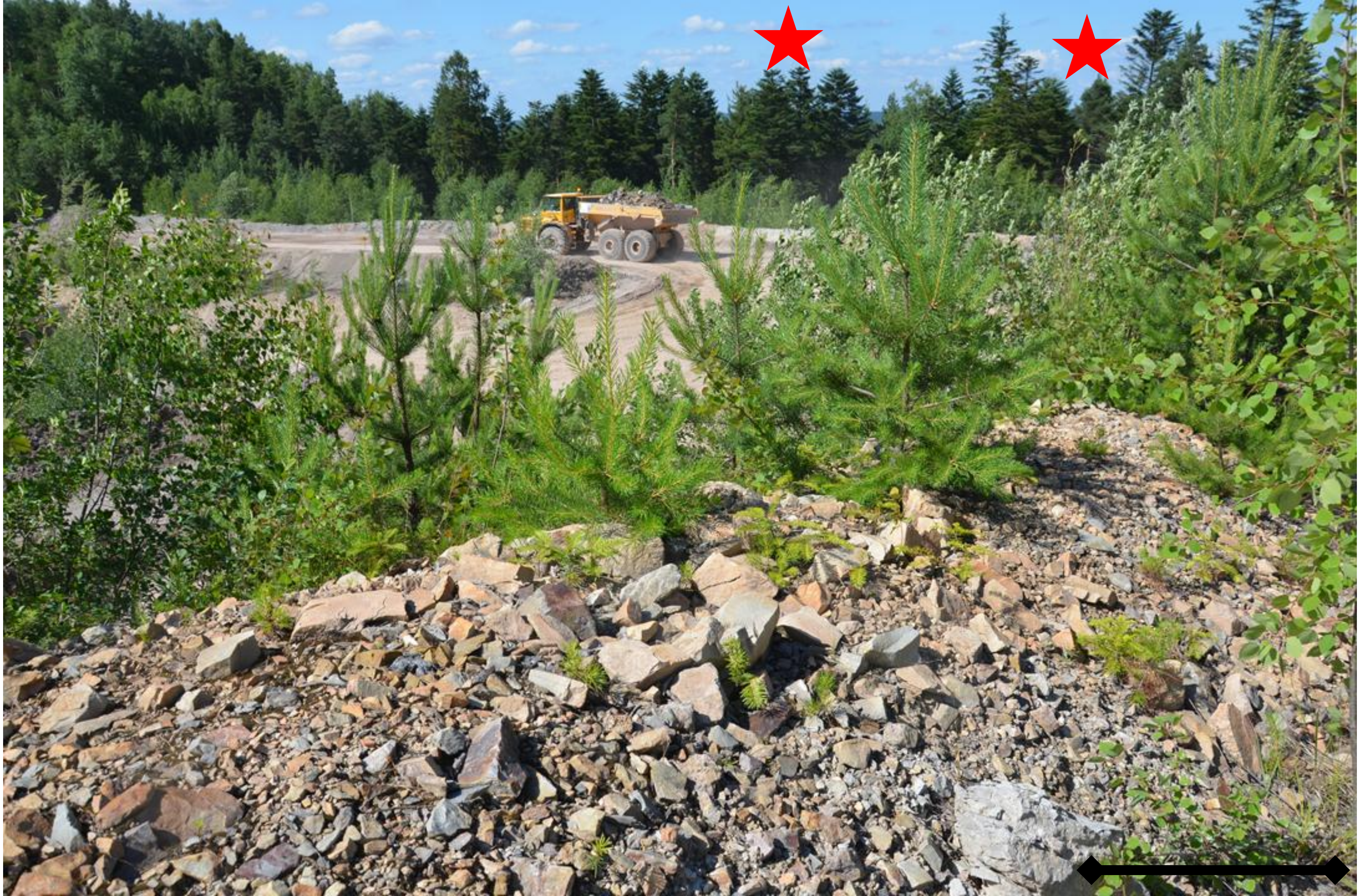

Supplement: Supplementary file 1 — Supplementary Material 1. Analyzed habitats and experimental sites - detailed data. [file 12870_2025_6792_MOESM1_ESM.pdf]
