# Supplementary material for: The high adaptive potential of Abies alba Mill. seedlings – biochemical and physiological studies of succession along the environmental gradient of a Cambrian quarry
Source: BMC Plant Biol. 2025 Jul 2;25:820. doi: 10.1186/s12870-025-06792-4 (PMC12220255; doi:10.1186/s12870-025-06792-4)
Supplement: Supplementary file 2 — Supplementary Material 2. Structural carbohydrates - FTIR results [file 12870_2025_6792_MOESM2_ESM.pdf]

The high adaptive potential of *Abies alba* Mill. seedlings – biochemical and physiological study of succession along the environmental gradient of cambrian quarry

Agnieszka Szuba, Ewelina Ratajczak, Tomasz Leski, Dominik Tomaszewski, Izabela Ratajczak, Gabriela Woźniak and Andrzej M. Jagodziński

Supplementary File S2

Structural carbohydrates – FTIR results

Structural carbohydrate FTIR results. Combined spectra for all six variants are presented. For clarity, comparisons of the four variants (which represented two habitats) are presented in one graph. Spectral comparison between needle-shaped seedlings collected from natural forest habitat (NFH) and disturbed forest habitat (DFH) (A) and between NFH and initial habitat (IH) (B). FTIR spectra comparison between the NFH stem and DFH (C) and between the NFH and IH (D). FTIR spectral comparison between NFH and DFH roots (E) and between NFH and IH roots (F). All the tree habitats are represented by two plots. The most important wavenumbers are marked with arrows (with thicker lines representing wavenumbers compared in Table 1). NFH – natural forest habitat; DFH – disturbed forest habitat; IH – initial habitat (for description, please see M&M section).

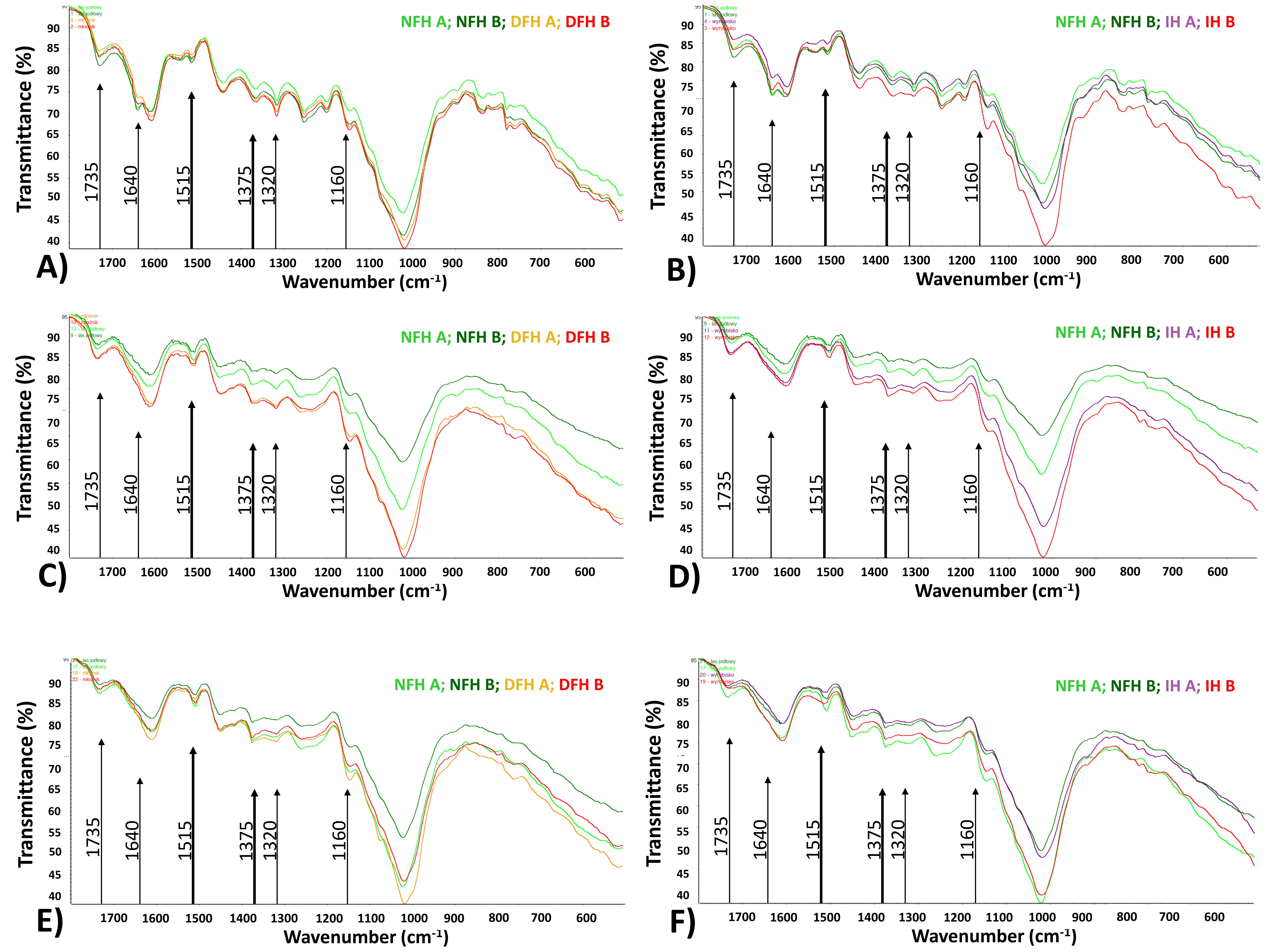

A)
NFH A; NFH B; DFH A; DFH B
Transmittance (%)
Wavenumber (cm⁻¹)
1735
1640
1515
1375
1320
1160
B)
NFH A; NFH B; IH A; IH B
Transmittance (%)
Wavenumber (cm⁻¹)
1735
1640
1515
1375
1320
1160
C)
NFH A; NFH B; DFH A; DFH B
Transmittance (%)
Wavenumber (cm⁻¹)
1735
1640
1515
1375
1320
1160
D)
NFH A; NFH B; IH A; IH B
Transmittance (%)
Wavenumber (cm⁻¹)
1735
1640
1515
1375
1320
1160
E)
NFH A; NFH B; DFH A; DFH B
Transmittance (%)
Wavenumber (cm⁻¹)
1735
1640
1515
1375
1320
1160
F)
NFH A; NFH B; IH A; IH B
Transmittance (%)
Wavenumber (cm⁻¹)
1735
1640
1515
1375
1320
1160
